# Structure of Bradavidin – C-Terminal Residues Act as Intrinsic Ligands

**DOI:** 10.1371/journal.pone.0035962

**Published:** 2012-05-04

**Authors:** Jenni Leppiniemi, Toni Grönroos, Juha A. E. Määttä, Mark S. Johnson, Markku S. Kulomaa, Vesa P. Hytönen, Tomi T. Airenne

**Affiliations:** 1 Institute of Biomedical Technology, University of Tampere, Tampere University Hospital, Tampere, Finland; 2 BioMediTech, Tampere, Finland; 3 Department of Biosciences, Biochemistry, Åbo Akademi University, Tykistökatu, Turku, Finland; Griffith University, Australia

## Abstract

Bradavidin is a homotetrameric biotin-binding protein from *Bradyrhizobium japonicum*, a nitrogen fixing and root nodule-forming symbiotic bacterium of the soybean. Wild-type (wt) bradavidin has 138 amino acid residues, whereas the C-terminally truncated core-bradavidin has only 118 residues. We have solved the X-ray structure of wt bradavidin and found that the C-terminal amino acids of each subunit were uniquely bound to the biotin-binding pocket of an adjacent subunit. The biotin-binding pocket occupying peptide (SEKLSNTK) was named “Brad-tag” and it serves as an intrinsic stabilizing ligand in wt bradavidin. The binding of Brad-tag to core-bradavidin was analysed by isothermal titration calorimetry and a binding affinity of ∼25 µM was measured. In order to study the potential of Brad-tag, a green fluorescent protein tagged with Brad-tag was prepared and successfully concentrated from a bacterial cell lysate using core-bradavidin-functionalized Sepharose resin.

## Introduction

Chicken egg-white avidin and its eukaryotic and prokaryotic homologs, known collectively as avidins, are proteins that have extreme affinity towards D-biotin (K_d_ ∼10^–15^ M for chicken avidin) [1]–[3]. Eukaryotic avidins exist in the eggs of e.g. birds, reptilia and amphibians [4]–[7], whereas prokaryotic avidins have been isolated from a few bacterial species: *Streptomyces avidinii* (streptavidin) [8], *Bradyrhizobium japonicum* (bradavidin and bradavidin II) [9], [10], *Rhizobium etli* (rhizavidin) [11] and *Burkholderia pseudomallei* (burkavidin) [12]. Avidins are known to be homotetrametric proteins, with the exception rhizavidin, which is a homodimer in its native form [11]. Due to the high-affinity interaction with D-biotin, avidins are widely applied in life sciences as well as in bio- and nanotechnology [13]–[15].

The best-studied avidins are the mature forms of chicken avidin and streptavidin. In the chicken egg-white, only the 28 amino acid signal peptide is removed from the full-length polypeptide chain to form the mature avidin protein (residues 1–128) [1], whereas several different cleavage products have been detected for streptavidin [16]. Streptavidin has been expressed as a recombinant protein with and without signal peptide [17], [18]. The most stable truncated form of streptavidin is the so-called core streptavidin, which contains residues 13–139 but still retains extremely high affinity towards D-biotin K_d_ ∼10^–15^ M [16]. Full-length wild-type (wt) streptavidin is expressed as a polypeptide of 159 residues. Interestingly, C-terminal residues 150–153 (Asn150-Gly151-Asn152-Pro153) of wt streptavidin fold back into the biotin-binding site in each monomer [19], thereby competing with the binding of at least low-affinity biotinylated macromolecules [16], [20].

Bradavidin is a tetrameric biotin-binding protein structurally and functionally resembling chicken avidin and other avidins. The gene encoding bradavidin was identified in *B. japonicum,* a nitrogen fixing and root nodule-forming symbiotic bacterium of the soybean [9]. Full-length, wt bradavidin has 138 amino acid residues, whereas the C-terminally truncated core form (core-bradavidin) has 118 amino acid residues [9]. Although bradavidin shares structural and functional similarities with other avidins, the percentage of amino acid sequence identity between the core regions of bradavidin and chicken avidin or streptavidin is only about 30%. As an additional indication of uniqueness, bradavidin has been proven to be immunologically different from chicken avidin and streptavidin [9]. Moreover, bradavidin has an acidic pI value (6.3 for wt bradavidin and 4.1 for core-bradavidin [9]) whereas mature chicken avidin (pI ∼10 [1]) is a basic protein. This property may allow the use of bradavidin instead of chicken avidin in applications disturbed by the charge-driven, non-specific binding of chicken avidin [21], [22].

In this study, we have determined the crystal structure of full-length, wt bradavidin at 1.8 Å resolution. Inspired by the X-ray structure, C-terminal residues occupying the biotin-binding pocket of wt bradavidin were evaluated as an affinity tag (Brad-tag) by using a synthetic peptide and by producing a fusion protein of enhanced green fluorescent protein (EGFP) and the Brad-tag. The binding of Brad-tag to core-bradavidin and other biotin-binding proteins were characterized using isothermal titration calorimetry (ITC) and its effect on the stability of bradavidin was determined by differential scanning calorimetry (DSC).

## Results

### The X-ray Structure of Bradavidin Reveals Unique Features

Wild-type bradavidin and core-bradavidin were produced in the periplasmic space of *E. coli* in an active form, essentially as previously described [9]. Bottle cultures (typical protein yields were around 1–5 mg/L) and a pilot-scale fermentor (yields of 3–7 mg/L) were used for protein expression. The isolated proteins were homogeneous and of high purity by SDS-PAGE analysis (data not shown).

**Figure 1:**
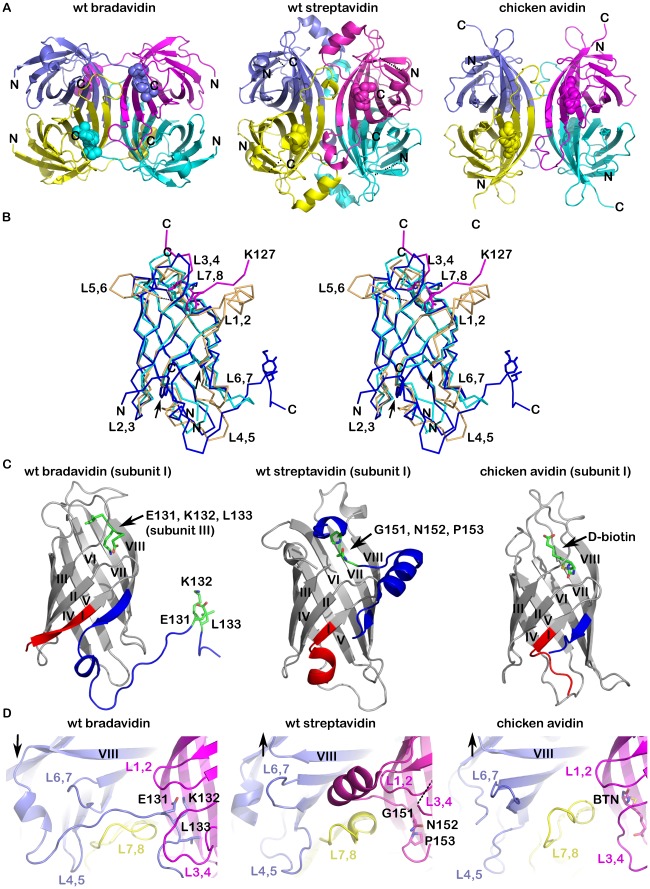
Structure comparison of wt bradavidin, wt streptavidin [PDB: 2BC3] and chicken avidin [PDB: 1AVD]. (**A**) Cartoon models of tetrameric proteins. Subunits are shown in different colors as follows: I (blue), II (cyan), III (magenta) and IV (yellow). The key biotin-binding pocket occupying residues (wt bradavidin and wt streptavidin) and biotin (chicken avidin) are shown as spheres. The N- and C-termini are indicated by letters. (**B**) Superimposition of the Cα traces of subunits I of wt bradavidin (blue), wt streptavidin (orange) and chicken avidin (cyan). The Cα trace for C-terminal residues starting from Lys127 of subunit III of wt bradavidin is shown, too. The ligand-binding site occupying residues Glu131-Leu133 of wt bradavidin and Gly151-Pro153 of wt streptavidin, and the biotin ligand of avidin, are shown as sticks. The left and right arrows pinpoint the N- and C-terminal sites, respectively, were major differences are seen between the proteins. Trp5 (left arrow) of wt bradavidin is shown as sticks. The N- and C-termini are indicated and loops are numbered. (**C**) Monomeric cartoon models. The N-terminus and C-terminus of each protein are indicated in red and blue, respectively, the colouring starting from equal positions in all proteins. The key residues occupying the biotin-binding site are shown as sticks. (**D**) Loop design. Colouring of subunits are as in (A) and representation of the key residues as in (C). The arrow pinpoints the varying beginnings of the L7,8-loops in the three structures. The L3,4-loop of the wt streptavidin structure is not fully visible (dashed line).

In order to help understand the molecular details behind the functional properties of bradavidin, the 3D structure of wt bradavidin was solved. We tried to crystallize wt bradavidin in the absence and presence of biotin, and in the presence of an azo dye HABA (4-hydroxyazobenzene-2-carboxylic acid, also called 2-(4′-hydroxybenzene)azobenzoic acid) in the hope that the dye would bind to the biotin-binding site. However, the protein crystallized only in the presence of HABA, but we could not identify HABA in the final structure. Orthorhombic crystals with a homotetramer in the asymmetric unit were obtained (for structure determination details, see Table 1). Each subunit I-IV (numbering according to [23]) of wt bradavidin had the overall β-barrel shape typical of avidins. A long C-terminal tail protruded from the closed-ends of the barrels and extended into the ligand-binding sites of the neighbouring subunits, serving as an intrinsic, intersubunit ligand (named Brad-tag; see below). More specifically, C-terminal residues of subunit I bound within the biotin-binding pocket of subunit III (and *vice versa*), and similar reciprocal interactions took place between subunits II and IV. These interactions uniquely anchored the ‘two dimers’ of the tetramer (a dimer of dimers) to each other (Figure 1A). This kind of intersubunit interaction has not been reported for any known member of the avidin family and thus provides an example of the utilization of an oligopeptide from adjacent subunits as an intrinsic ligand. In the X-ray structure of the T7-tagged wt streptavidin, C-terminal residues 150–153 also occupy the biotin-binding site [19], however, the extended C-terminus and the ligand-binding pocket into which it folds is formed by a single polypeptide chain (intrasubunit ligand). The overall intrinsic ligand-binding architecture is therefore clearly different in bradavidin and in streptavidin (Figure 1).

**Table 1:** X-ray structure determination statistics for wt bradavidin [PDB: 2Y32].

Cell parameters	
Space group	*P*2_1_2_1_2_1_
Unit cell:	
a, b, c (Å)	46.7, 84.9, 120.3
α, β, γ (°)	90, 90, 90
**Data collection** a	
Wavelength (Å)	1.04192
Beamline	I911–2 (MAX-lab, Lund)
Detector	MarCCD
Resolution (Å)b	25–1.78 (1.78–1.88)
Unique observations^b^	46670 (6955)
I/sigma^b^	18.0 (5.9)
*R*_factor_ (%)^b^	9.0 (42.4)
Completeness^b^	100 (100)
Redundancy^b^	9.6 (9.6)
**Refinement**	
*R*_work_ (%)^c^	14.4
*R*_free_ (%)^c^	18.2
Monomers (asymmetric unit)	4
Protein atoms	4115
Solvent atoms	680
*R.m.s.d:*	
Bond lengths (Å)	0.014
Bond angles (°)	1.4

a The numbers in parenthesis refer to the highest resolution bin.

b From XDS [53].

c From Refmac 5 [57] using TLS [71] & restrained refinement.

**Figure 2:**
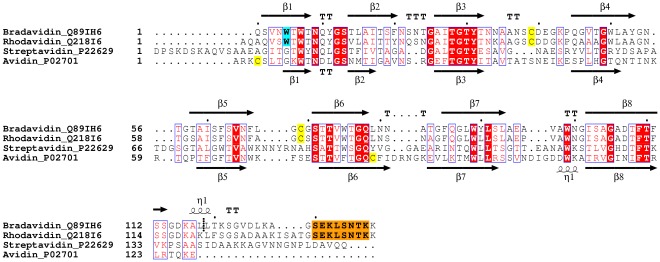
Sequence alignment of wt bradavidin, rhodavidin, streptavidin and chicken avidin. The UniProt [70] accession numbers are shown after the names of the sequences. The Brad-tag sequence is highlighted with orange background, cysteine residues with yellow and the tryptophan residue important for the N-terminus of wt bradavidin with cyan. The secondary structures of wt bradavidin (from the structure reported here) and chicken avidin (from [PDB: 1AVD]) are shown. The truncation site for core-bradavidin is between Leu118 and Leu119 and is shown by a short, vertical dashed line. The conserved residues are indicated by the default colouring scheme of the ESPript program. TT, β-turn; TTT, α-turn; and η1, 3/10-helix. The structural alignment was created in Bodil [66] and the picture using ESPript [68].

Even though the quaternary structure of wt bradavidin resembles the known structures of avidins (Figure 1A), some of the secondary structure elements and loops, especially those in the close proximity to either the N- or C-terminal residues, differ noticeably in comparison to other avidins (Figures 1B, 1C, 2). For example, the first five amino acids of wt bradavidin have different spatial locations in comparison to wt streptavidin or chicken avidin. Residue Trp5 of bradavidin is located in a key position and helps determine the unique conformation of the N-terminus: the Trp5 side chain is located in a hydrophobic pocket created by residues that include Val3, Trp7, Ile17, Ile27, Leu51, Phe20, Leu118 and Leu119; the side-chain nitrogen atom of Trp5 is hydrogen bonded to the carbonyl oxygen of Asp115, too. Residues 1–3 of wt bradavidin interact with residues 17–20 of the β2-strand and the adjacent L2,3-loop. Additionally, the side-chain oxygen atom of Asn4 is hydrogen bonded to the backbone nitrogen atom of Asp115, located at the beginning of the C-terminal extension. Unique structural features of the C-terminus of wt bradavidin are seen beginning with residues directly following β-strand 8, and extending through the adjacent short 3/10-helix and then turning towards and entering the neighbouring subunit (Figure 1D). Ala128 and Gly129 are the first residues clearly leaving the original subunit and form a linker between the original (e.g. subunit I) and the neighbouring subunit (e.g. subunit III), whereas the terminal residues 130–138 are part of the neighbouring subunit. Together, the N- and C-terminal residues have a clear effect on determining the shape of the fold of wt bradavidin. In bradavidin, the L4,5-loop and the L6,7-loop of one subunit seem to be adapted to pack and guide the C-terminal extension towards the neighbouring subunit and the L7,8-loop of neighbouring subunit contributes to the conformation of the C-terminus, too (Figure 1D). Several of the β-strands, i.e. strands 1, 6 and 8, also differ in their spatial arrangements (length and/or orientation) in comparison to chicken avidin and streptavidin. Moreover, analysis of the surface of the bradavidin structure identified bradavidin-specific characteristics (Figure S1).

### Subunit Interfaces of Bradavidin

In a previous study we found that bradavidin in the presence of biotin was structurally less stable when compared to either chicken avidin or streptavidin [9]. Avidins gain stability via oligomerization [24], [25] and the subunit-interfaces play a significant role in determining their stability. Consequently, the subunit-subunit contacts of bradavidin were carefully examined.

**Figure 3:**
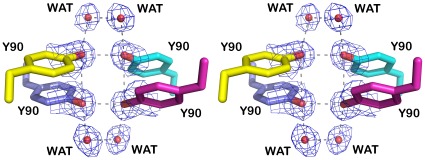
Four tyrosine residues at the intersection of all four subunit interfaces in bradavidin. A stereo view. The tyrosine residues are shown as stick models with different colouring for the different subunits (subunit I, blue; II, cyan; III, magenta; and IV, yellow). Structural water molecules are shown as red spheres. Electron density map (a weighted 2FO-FC map; sigma level 1) around the water molecules and the side chain oxygen atoms of Tyr90 is shown in blue. Putative hydrogen bonds are indicated with grey dashes.

Four tyrosine residues (Tyr90), one from each subunit, are located in the center of the bradavidin tetramer (Figure 3), and they are likely to play a major role in the assembly and stability of the tetramer. Structural water molecules in the vicinity (2.6–2.8 Å) of the hydroxyl groups support the idea that hydrogen bonds together with ring stacking stabilize the tetramer.

In bradavidin, there are eight residues (Trp89, Tyr90, Leu91, Trp99, Asn100, Ile102, Ser103 and Ala104) having non-hydrogen atoms within 4 Å of each other at the interface of subunits I and II. Ile102, Ser103 and Ala104 form the core of the I-II interface and, together with Tyr90 and Asn100, are the most characteristic residues of the I-II subunit interface (Figure S2). Of these residues, only an isoleucine equivalent to Ile102 of bradavidin is found in one other known avidin structure, rhizavidin [11]. Interactions among residues (102–104) of the I-II interface include conventional hydrogen bonds between the backbone oxygen atom of Ile102 and the backbone nitrogen atom of Ala104– these backbone interactions are well conserved within the avidin family. A weak hydrogen bond (C-H^…^O; [26], [27]) between the side-chain oxygen atom of Ser103 and the Cα atom of Asn100 was also seen. Moreover, the Cη atom of Trp89 is at a distance of 3.6 Å, and thus able to form a weak hydrogen bond (C–H^…^O) with the backbone oxygen atom of Trp99. Both Trp89 and Trp99 are well conserved within the avidin family, including chicken avidin and streptavidin, and are known to line the biotin-binding pocket. Leu91 of bradavidin is also well conserved and its side chain is involved in van der Waals interactions with the side chain of Trp99. The side chain of Tyr90 is in van der Waals contact with the side-chain atom of Ala104.

The core of the I-III subunit interface of wt bradavidin is formed by Gln86, Leu88, Tyr90, Ala104 and Ala106 (Figure S2), which are all poorly conserved in the avidin family. For example, Gln86, Leu88, Tyr90, Ala104 and Ala106 in bradavidin are in chicken avidin (and streptavidin) Lys94 (Asn105), Met96 (Gln107), Leu98 (Leu109), Val115 (Val125) and Ile117 (His127). The nitrogen atom of the Gln86 side chain of subunit I is hydrogen bonded with the carbonyl oxygen atom of Ala106 of subunit III (and vice versa), and the Cβ atoms of Ala106 of subunits I and III are in van der Waals contact with each other. Weak van der Waals interactions between the side chain of Leu88 and the atoms of Ala104 are apparent, too. Tyr90 of subunit I is within hydrogen-bonding distance (2.8 Å) of Tyr90 of subunit III. The C-terminal residues 129–137, containing the residues of the Brad-tag, are also involved in I-III subunit interactions but these residues are described separately below.

The I-IV subunit interface of bradavidin involves 47 amino acid residues (4 Å probe using non-hydrogen atoms) and is stabilized by a large number of non-covalent interactions. In comparison to known avidin structures, the I–IV subunit interface of bradavidin is also distinctive. In addition to the central Tyr90 and the C-terminal Brad-tag, the I–IV subunit interface is stabilized by van der Waals interactions and hydrogen bonding of e.g. the side chain of Trp50 with the atoms of residues Gly46, Thr48, Ser63, Val64 and Asn65, and the side chain of the well-conserved Gln78 is uniquely packed against residues of the L7,8-loop, and Trp99 of this loop is part of the ligand-binding site.

All in all, tetrameric wt bradavidin is stabilized by a large set of non-covalent intersubunit interactions, many of which are unique to bradavidin within the members of the avidin family. However, the decreased thermal stability of bradavidin suggests that the subunit interfaces of wt bradavidin are less optimal for maintaining the tetrameric stability as in chicken avidin or streptavidin.

### The Ligand-binding Site of wt Bradavidin has an Intrinsic Ligand

**Figure 4:**
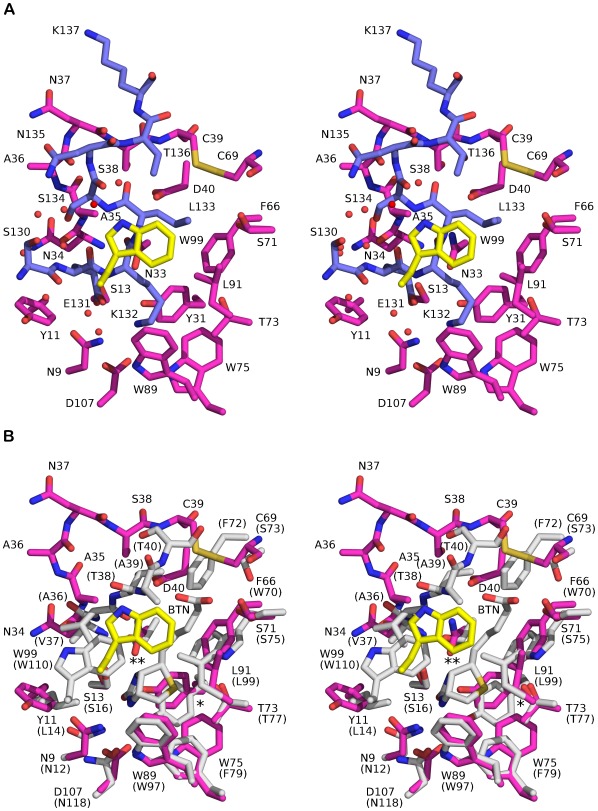
Stick model (stereo view) of the ligand-binding site of wt bradavidin. (**A**) The carbon atoms of the Brad-tag sequence (subunit I) are shown in blue and the residues around the Brad-tag sequence in magenta (subunit III) and yellow (subunit IV). Structural water molecules near the Brad-tag sequence are shown as red spheres. (**B**) Superimpositioning of the ligand-binding site of wt bradavidin and chicken avidin [PDB: 1AVD]. Colouring for wt bradavidin as in (A); the carbon atoms of residues of chicken avidin are shown in grey. The residues Asn12, Leu14, Ser16, Tyr33, Trp70, Ser73, Ser75, Thr77, Phe79, Trp97, Leu99 and Asn118 of chicken avidin were superimposed to the equivalent residues Asn9, Tyr11, Ser13, Tyr31, Phe66, Cys69, Ser71, Thr73, Trp75, Trp89, Leu91 and Asp107 of wt bradavidin. For clarity, the Brad-tag sequence is not shown. For chicken avidin, the residue numbers are shown in brackets. BTN, biotin; *, Tyr31 (Tyr33); **, Asn33 (Thr35).

The C-terminal extension of each subunit in wt bradavidin reaches out and occupies the ligand-binding site of an adjacent subunit, serving as an intrinsic, intersubunit ligand. Namely, residues 130-138 of subunits I and II respectively occupy the ligand-binding pocket of subunits III and IV, and vice versa (Figure 1). In comparison to chicken avidin [PDB: 1AVD; [28]] or wt streptavidin [PDB: 2BC3; with a T7-tag], the ligand-binding site of wt bradavidin is rich in novel features. For example (see Figure 4A), the C-terminus (Ser130-Lys137) of subunit I has two consecutive turns mimicking the shape of an S letter (“S-shaped”) and embedding between Ser13, Tyr31 and Asn33-Asp40 (L3,4-loop) of the neighbouring subunit III on one side and, on the other side, Trp99 of a third subunit (IV). The binding pocket (III) is lined by Asn9, Tyr11, Phe66, Ser71, Thr73, Trp75, Trp89, Leu91 and Asp107 and the L3,4-loop is stabilized by an intrasubunit disulfide bond between Cys39 and Cys69, similar to rhizavidin [29]. In general, the C-terminus causes the opening or widening of the ligand-binding pocket of wt bradavidin in a manner not observed for any other known avidin structure. The binding of the C-terminal extension of bradavidin is stabilized by a number of non-covalent interactions and several structural water molecules are found in close proximity of the binding site (Figure 4A).

Glu131, Lys132 and Leu133 of wt bradavidin insert deepest into the ligand-binding pocket of the adjacent subunit. When superimposed with chicken avidin [PDB: 1AVD], it is evident that residues 131-133 occupy the site equivalent to the biotin-binding site of avidin (Figure S3). The Leu133 side chain matches the location of the carboxylate end of biotin, whereas the side chain of Lys132 fills the space occupied by the bicyclic ring system (2′-keto-3,4-imidazolidotetrahydrothiophene moiety) of biotin. Glu131 stabilizes the local structure, and especially, the side-chain conformation of Lys132 (Figure S4). With respect to the wt streptavidin structure [PDB: 2BC3], Leu133 superposes with Pro153, whereas the side chain of Lys132 overlaps with Gly151. Even though some of the residues lining the ligand-binding site of bradavidin, avidin and streptavidin are identical in sequence and are structurally conserved (Figure 4B), the binding mode of Glu131-Lys132-Leu133 is different from that seen for Gly151-Asn152-Pro153 in the wt streptavidin structure and for biotin in the avidin structure (Figures 4, S3). This is due to the two-layered, “S-shaped” conformation of the C-terminus of bradavidin, the terminus entering into a neighbouring subunit (Figures 1, 4). Moreover, the side-chain nitrogen atom of K132 is stabilized by several hydrogen bonds; interaction of K132 (NZ atom) with the side-chain oxygen atoms of Y31, D107 and E131 are possible; Y31 and D107 are from a different subunit (Figure S4).

To get an idea how biotin would bind to the presumed ligand-binding site of wt bradavidin and how it would affect to the conformation of the C-terminus, we tried to co-crystallize wt bradavidin with biotin. However, we did not get any crystals with biotin. Therefore, selected residues lining the biotin-binding site of chicken avidin [PDB: 1AVD] were superimposed with the equivalent residues of wt bradavidin (Figure 4B). We left out the C-terminal residues (Brad-tag) of wt bradavidin from the structural comparison because of two reasons: 1) the C-terminus is very likely to undergo major conformational changes due to biotin binding, changes that would require heavy computations to give reasonable predictions (beyond this study); and 2) direct contacts between the C-terminal residues and biotin are not likely to occur. Our structural comparison suggests that the L3,4-loop of wt bradavidin, and especially the residues Ala35-Ser38 of it, should undergo a major conformational change for efficient biotin binding, whereas most of the other residues (i.e. not in the L3,4-loop) lining the presumed biotin-binding site of wt bradavidin have conformations close to those seen in chicken avidin and are hence likely to undergo only minor conformational changes due to biotin binding. In conclusion, most of the interactions seen between chicken avidin and biotin [PDB: 1AVD] are probably also seen in wt bradavidin when in complex with biotin.

Several peptide tags binding to the biotin-binding site of avidin, and particularly to streptavidin, have been reported (Table 2), and X-ray structures are known for the Strep-tag [PDB: 1RST], Strep-tagII [PDB: 1RSU, 1KL3/5] and Nano-tag [PDB: 2G5L] streptavidin complexes. However, the binding modes of these tags are clearly different from that seen for bradavidin (Figure S5). For example, the peptide backbone of Strep-tag [30], Strept-tag II [30], [31] and Nano-tag [32] have a 3_10_-helical conformation, whereas the C-terminus of wt bradavidin is a combination of two consecutive turns.

**Table 2:** Summary of different avidin-binding peptide tags.

Tag	Sequence(residues)	Size(kDa)	Receptor	K_d_	PDBentry	References
Avi-tag	DRATPY (6)	0.72	Avidin,NeutrAvidin	28 µM12 µM	a	[72]
AviD-tag	Divalent DRATPY(6+spacer+6)	1.44+spacer	Avidin,NeutrAvidin	a	a	[73]
Nano-tag_15_	fMDVEAWLGARVPLVET(formyl-Met+15)	1.81	Streptavidin	4 nM	a	[74]
Nano-tag_9_	fMDVEAWLGAR (formyl-Met+9)	1.18	Streptavidin	17 nM	a	[74]
Nano-tag	fMDVEAWL(formyl-Met+6)	0.89	Streptavidin	<20 nM	2G5L	[32]
Strep-tag	AWRHPQFGG (9)	1.06	Streptavidin	37 µM	1RST	[30]
Strep-tag II	WSHPQFEK (8)	1.06	StreptavidinSA mutant 1 i.e. StrepTactinSA mutant 2	72 µM1 µM1 µM	1RSU 1KL31KL5	[30], [31], [75]
SBP-tag	MDEKTTGWRGGHVVEGLAGELEQLRARLEHHPQGQREP (38)	4.31	Streptavidin	2.5 nM	a	[76]
Brad-tag	SEKLSNTK (8)	0.91	Core-bradavidin	25 µM		This study

a Not available.

### Isothermal Titration Calorimetry Reveals Moderate Affinity between Brad-tag and Core-bradavidin

**Figure 5:**
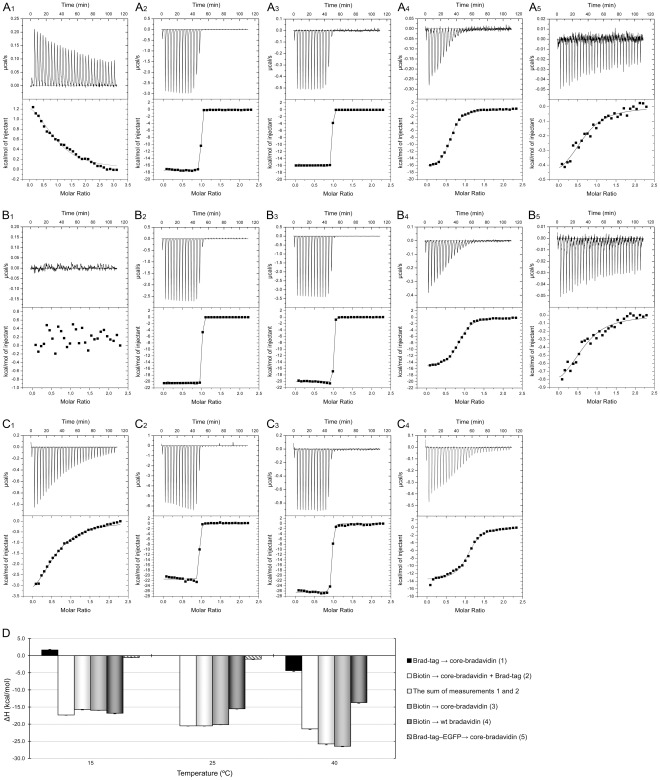
ITC analysis of ligand binding. Thermograms of measurements performed at three different temperatures (**A**) 15°C, (**B**) 25°C and (**C**) 40°C are shown. At each temperature, core-bradavidin was first titrated with Brad-tag (1), followed by competitive titration with biotin (2). As a control measurement, core-bradavidin was titrated with biotin only (3). In order to prove that the intrinsic Brad-tag decreases the affinity towards biotin, wt bradavidin was also titrated with biotin (4). In addition, core-bradavidin was titrated with Brad-tag–EGFP at 15 and 25°C (5). (**D**) Comparison of the binding enthalpies of all measurements at different temperatures. Brad-tag had a clear effect on the binding enthalpy of the competitive titration with biotin at 15°C (endothermic Brad-tag binding) and 40°C (exothermic Brad-tag binding). At 25°C, the enthalpy of competitive titration was equal to that of titration with biotin only (no detectable binding of Brad-tag to core-bradavidin).

We used isothermal titration calorimetry (ITC) to determine the affinity between core-bradavidin and a synthetically produced Brad-tag (SEKLSNTK), and found that the binding enthalpy of Brad-tag to core-bradavidin was temperature dependent (Figure 5, Table 3). At 15°C, the enthalpy was found to be positive (1.1±0.2 kcal/mol, Figure 5A_1_) indicating endothermic binding, whereas a negative binding enthalpy (–4.4±0.3 kcal/mol, Figure 5C_1_) was observed for core-bradavidin titrated with Brad-tag at 40°C, indicating the exothermic nature of binding. At 25°C, the binding enthalpy was negligible (Figure 5B_1_) most likely because the measurement was performed close to the transition temperature between the exothermic and endothermic binding mode. The calculated dissociation constant (*K_d_*) was 25 µM at 15°C and 26 µM at 40°C but could not be determined at 25°C.

**Table 3:** Thermodynamic parameters of ligand binding analyzed by ITC.

Cell		Syringe		T	ΔH	-TΔS	ΔG	K_d_	n
Contents	c_initial_	Contents	c_initial_						
	[mM]		[mM]	[°C]	[kcal mol^–1^]	[kcal mol^–1^]	[kcal mol^–1^]	[M]	
Core-bradavidin	0.073	Brad-tag	0.985	15	1.7±0.2	–7.7	–6.1	2.5±0.5×10^–5^	0.94
Brad-tag/core-bradavidin	0.169/0.06	Biotin	0.6	15	–17.3±0.0	ND^a^	ND^a^	<10^–9^	0.95
Core-bradavidin	0.01	Biotin	0.1	15	–15.9±0.1	ND^a^	ND^a^	<10^–9^	0.93
Core-bradavidin	0.015	Brad-tag–EGFP	0.15	15	–0.5±0.1	–6.8	–7.3	2.8±0.8×10^–6^ b	0.68 b
wt Bradavidin	0.01	Biotin	0.1	15	–16.8±0.2	8.1	–8.7	2.7±0.2×10^–7^	0.61
Core-bradavidin	0.05	Brad-tag	0.25	25	ND^c^	ND^c^	ND^c^	ND^c^	ND^c^
Brad-tag/core-bradavidin	0.043/0.041	Biotin	0.4	25	–20.5±0.1	ND^a^	ND^a^	<10^–9^	0.97
Core-bradavidin	0.05	Biotin	0.5	25	–20.1±0.1	ND^a^	ND^a^	<10^–9^	0.98
Core-bradavidin	0.015	Brad-tag–EGFP	0.15	25	–1.0±0.1	–6.5	–7.5	3.1±1.0×10^–6^ b	0.68 b
wt Bradavidin	0.01	Biotin	0.1	25	–15.5±0.1	6.6	–8.9	3.0±0.2×10^–7^	0.85
Core-bradavidin	0.1	Brad-tag	1	40	–4.4±0.3	–2.2	–6.6	2.6±0.3×10^–5^	0.62
Brad-tag/core-bradavidin	0.172/0.083	Biotin	0.8	40	–21.4±0.1	ND^a^	ND^a^	<10^–9^	0.91
Core-bradavidin	0.01	Biotin	0.1	40	–26.4±0.1	ND^a^	ND^a^	<10^–9^	0.93
wt Bradavidin	0.01	Biotin	0.1	40	–13.7±0.2	4.3	–9.4	2.6±0.4×10^–7^	1.08

a K_d_ could not be determined as it exceeded the sensitivity limit of ITC.

b A rough estimate because the noise level in thermogram was relatively high.

c Could not be determined.

We could not determine the *K_d_* of D-biotin for core-bradavidin, as the measurement exceeded the sensitivity limit of ITC (*K_d_* <10^–9^M). However, ITC titration provided the enthalpy and stoichiometry of the binding. The highly negative binding enthalpies (–15.9±0.1, –20.1±0.1, and –26.4±0.1 kcal/mol at 15, 25 and 40°C, Figures 5A_3_, B_3_, C_3_, respectively) indicated the exothermic nature of biotin binding and were in accordance with our earlier observations with rhizavidin [11] and avidin [33]. The average of the calculated stoichiometries of binding at different temperatures was 0.78±0.23/subunit, suggesting 1∶1 binding of Brad-tag to core-bradavidin subunits. In a competitive binding assay, core-bradavidin was first saturated with Brad-tag followed by a second titration with biotin (Figures 5A_2_, B_2_, C_2_). At 15°C, the measured enthalpy (–17.3±0.1 kcal/mol) was more negative in the competitive binding assay than in the non-competitive assay, because core-bradavidin was saturated with Brad-tag (positive enthalpy) before titration with biotin (Figure 5D). At 25°C, the enthalpies for competitive and non-competitive binding of biotin differed only by 0.4 kcal/mol; the enthalpy measured for the binding of Brad-tag to core-bradavidin was close to zero at this temperature. At 40°C, the competitive biotin titration resulted in less negative enthalpy (–21.4±0.1 kcal/mol) than the non-competitive binding. The sum of the measured binding enthalpy of core-bradavidin titrated with Brad-tag and the enthalpy of the competitive titration with biotin was almost equal to the binding enthalpy of core-bradavidin titrated with biotin only.

Avidin, streptavidin and rhizavidin were titrated with Brad-tag at 40°C (Figure S6) in order to study the specificity of Brad-tag towards core-bradavidin. No sign of interaction was observed, indicating that Brad-tag is specific for core-bradavidin.

### C-terminal Extension Stabilizes wt Bradavidin and Inhibits Biotin Binding

ITC analysis revealed a clearly reduced biotin-binding affinity for wt bradavidin (*K_d_* ∼2.8×10^–7^ M) compared to core-bradavidin (*K_d_* <10^–9^ M) (Figure 5, Table 3). This indicates that the C-terminal extension of bradavidin competes with biotin for the ligand-binding site, which is in agreement with the structure of wt bradavidin. The exothermic binding was detected as highly negative enthalpies (–16.8±0.2, –15.5±0.1, and –13.7±0.2 kcal/mol at 15, 25 and 40°C; Figures 5A_4_, B_4_, C_4_, respectively). At 40°C, we observed a two-phase binding-curve and the non-linear least-squares fit was not perfect over the first three titration steps, increasing the error in the binding enthalpy. One possible explanation for this could be that the C-terminus becomes more flexible at 40°C due to increased thermal motion.

Differential scanning calorimetry (DSC) was used to study the heat-induced unfolding of core-bradavidin and wt bradavidin. The melting temperatures (T_m_) for core-bradavidin in the absence and in the presence of biotin were 73.2±0.3°C and 97.9±0.2°C. For wt bradavidin, the T_m_ value without biotin was 96.2±0.1°C, and 101.7±0.1°C with biotin. These results further indicate that the C-terminal extension (including the Brad-tag) can serve as an intrinsic ligand and stabilize wt bradavidin, since the melting temperatures of wt bradavidin with and without biotin were quite similar. No peaks were seen in the second heating scan of DSC analysis, indicating irreversible unfolding, which is typical for avidin proteins.

### Feasibility of Brad-tag as Affinity Tag

**Figure 6:**
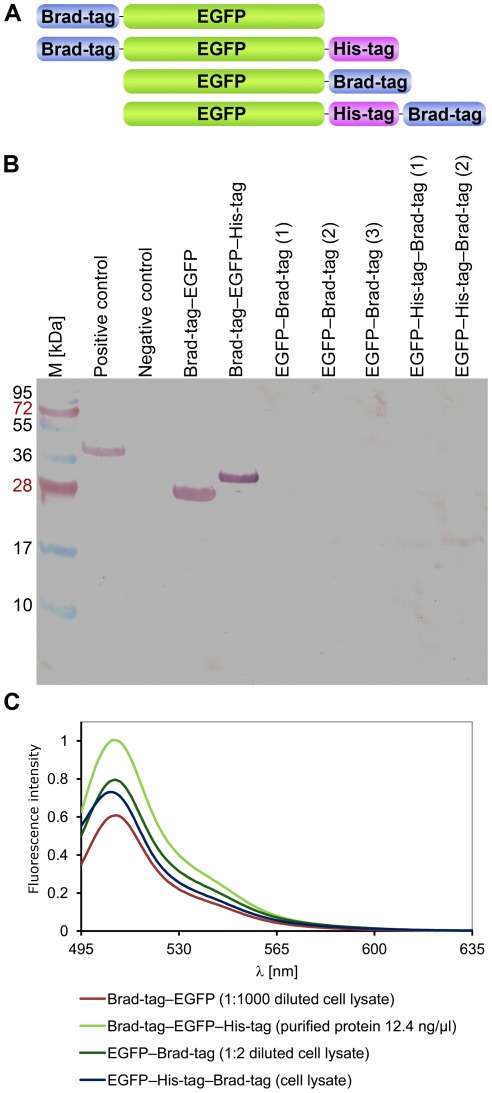
Brad-tag–EGFP fusion proteins. (**A**) Four different Brad-tag–EGFP fusion protein constructs were used in the current study. Brad-tag was positioned at the N- or C-terminus of the fusion proteins. A His-tag was also included in two of the constructs directly after the sequence of EGFP. (**B**) Immunoblot analysis using antibody against GFP was used to evaluate the quality and amount of tagged EGFPs. Biotinylated–EGFP was used as a positive control (Vikholm-Lundin et al, unpublished) and core-bradavidin as a negative control. Numbers in brackets indicate different protein productions. Molecular weight markers (M, kDa) are indicated on the left. (**C**) The fluorescence spectra measured for purified Brad-tag–EGFP–His-tag (12.4 ng/µl) and clarified cellular lysates of other Brad-tag–EGFP constructs.

In order to study the potential of Brad-tag as an affinity tag, we designed four different fusion proteins with EGFP as described in Figure 6A. The recombinant Brad-tag–EGFP fusion proteins were produced in *E. coli* and their expression was analyzed by immunoblotting, where Brad-tag–EGFP and Brad-tag–EGFP–His-tag were clearly recognized by an antibody against GFP (Figure 6B). In contrast, the signal from C-terminally tagged EGFPs was negligible, indicating that proteins were not produced efficiently. Shorter protein forms were also seen in the lysates corresponding to C-terminally tagged EGFPs, possibly resulting from proteolytic cleavage. The spectrofluorometric analysis of cellular lysates, however, confirmed that the C-terminally tagged EGFPs were expressed (Figure 6C).

**Figure 7:**
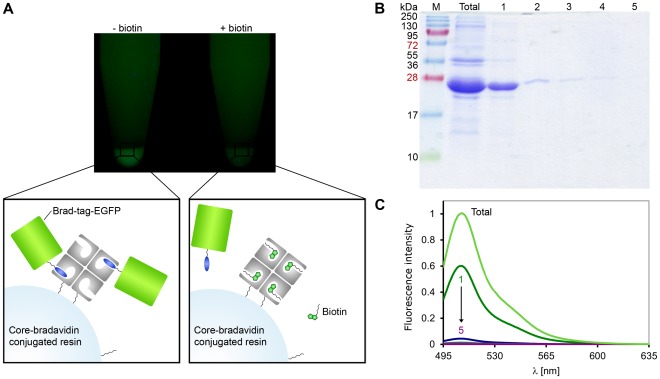
Purification of EGFP fusion protein using N-terminal Brad-tag. (**A**) Photograph of Brad-tag–EGFP–His-tag bound to core-bradavidin resin under UV-light. First, core-bradavidin was coupled by amine groups to the terminal NHS carboxylates of the linkers (resin–NH–(CH_2_)_5_–COONHS). Then, Brad-tag–EGFP–His-tag (prepurified with Ni-NTA column) was incubated with the functionalized resin and the resin was pelleted. In the absence (–) of biotin Brad-tag–EGFP–His-tag concentrates on the resin pellet. The presence (+) of free biotin inhibits the binding. Stoichiometry and the size of compounds in the schematic figure are only speculative. (**B**) SDS-PAGE analysis of the protein purification experiment for cellular lysates of N-Brad-tag–EGFP-C. Cleared cellular lysate (total) was incubated with core-bradavidin resin. Then the resin was washed with buffer (50 mM Tris-HCl, 100 mM NaCl, pH 7.5) and samples 1 to 5 were eluted. Molecular weight markers (M, kDa) are indicated on the left. (**C**) The fluorescence spectra measured for cleared cellular lysate (total) and eluted samples 1 to 5 from the protein purification experiment for Brad-tag–EGFP.

To find out whether the Brad-tag would prove functional in protein purification, core-bradavidin was conjugated to a sepharose resin. As a first experiment, Ni-NTA purified Brad-tag–EGFP–His-tag fusion protein was loaded onto the core-bradavidin-conjugated resin, which concentrated the fusion protein (Figure 7A). In contrast, a resin saturated previously with free biotin had no concentrating effect showing that biotin can efficiently prevent Brad-tag–core-bradavidin interaction. As a second experiment, we also studied whether Brad-tag–EGFP could be purified from cleared cellular lysates with core-bradavidin-conjugated resin. First, cleared cellular lysates were incubated with resin, and then the resin was washed with the binding buffer, which quite rapidly (∼5–10 column volumes) led to dissociation of the bound protein. The protocol still made it possible to separate Brad-tagged EGFP from other proteins of the cell lysate as verified by SDS-PAGE (Figure 7B). Moreover, the emission spectrum of the isolated Brad-tag–EGFP protein strongly resembled that of free EGFP indicating that the tag did not affect the conformation of EGFP (Figure 7C).

In order to further study the functionality of Brad-tag, we performed ITC analysis using Brad-tag–EGFP and core-bradavidin. Here, slightly negative binding enthalpies (–0.5±0.1 at 15°C and –1.0±0.1 kcal/mol at 25°C) were detected, indicating exothermic binding. However, the noise level in the thermograms (Figures 5A_5_, B_5_) was relatively high because of low protein concentrations, and therefore, the non-linear least-squares fit only gave a very rough estimate of the *K_d_* : ∼3.0×10^–6^ M (Table 3).

### Specificity of Brad-tag Binding

**Figure 8:**
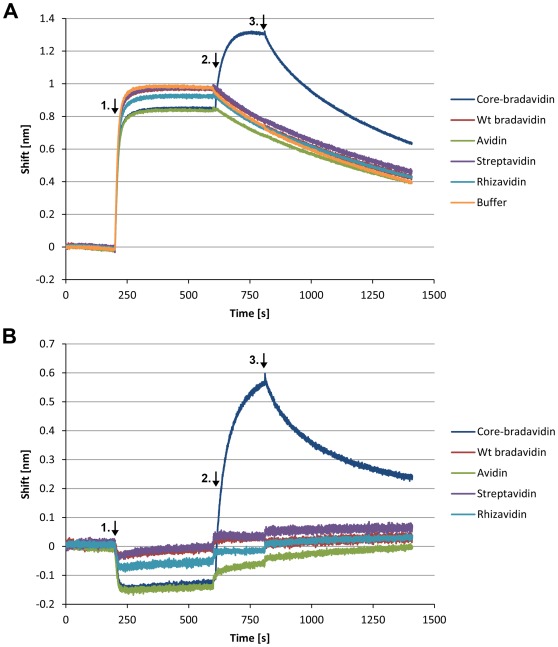
Specificity of core-bradavidin binding to Brad-tag analyzed by biolayer interferometry. (**A**) Anti-Penta-HIS biosensors were functionalized with Brad-tag–EGFP–His-tag fusion protein (step 1, arrow in the graph). After a brief wash (10 s) in measurement buffer, biosensors were incubated with a series of different avidin proteins at concentration of 0.06 mg/ml (step 2). Sample without any avidin protein was used as a negative control (buffer). Finally, biosensors were exposed to the measurement buffer leading to dissociation of the bound core-bradavidin proteins (step 3). (**B**) The measured raw data for buffer is subtracted from the raw data of different proteins.

The specificity of core-bradavidin binding to Brad-tag was studied using a biolayer interferometry biosensor ForteBio Octet RED384. Anti-Penta-HIS biosensors were functionalized with Brad-tag–EGFP–His-tag fusion protein showing efficient binding (Figure 8A, shift ∼1 nm). The Brad-tag-functionalized sensors were then incubated in the presence of different avidin proteins. At a protein concentration of 0.06 mg/ml, only core-bradavidin showed clear binding to the sensor surface, whereas wt bradavidin, avidin, streptavidin or rhizavidin were not distinguishable from a sample with plain buffer (Figure 8A). We noticed dissociation of Brad-tag–EGFP–His-tag from the sensor surface, and therefore the buffer sample was subtracted from the other measured data to better illustrate the binding reaction (Figure 8B).

To further prove the specificity of the binding (Figure S7), we exposed the Brad-tag–EGFP–His-tag functionalized sensor to solutions containing higher protein concentrations as follows: core-bradavidin (0.5 mg/ml), wt bradavidin (1.2 mg/ml), chicken avidin (1.8 mg/ml), streptavidin (1.7 mg/ml) and rhizavidin (2.0 mg/ml). Core-bradavidin showed a strong binding response (shift 1.2 nm, Figure S7A), and chicken avidin showed a small response (shift 0.13 nm; Figure S7A). However, further control measurements revealed that chicken avidin bound equally well to plain anti-Penta-HIS biosensors (shift ∼0.35 nm, Figure S7D). This was not surprising, as avidin is a highly basic protein (pI ∼10) [1], and has in numerous previous studies been shown to bind non-specifically on different materials. Interestingly though, the binding of chicken avidin to both sensor types could be inhibited in the presence of free biotin, which seems to be the case also for chicken avidin–DNA interaction [34]. Core-bradavidin also showed some binding to non-functionalized anti-Penta-HIS sensors (shift ∼0.45 nm; Figure S7D), but this binding was not dependent on the presence of biotin. Importantly, core-bradavidin showed no binding to the Brad-tag–EGFP–His-tag-functionalized surface in the presence of biotin (Figure S7C), thus proving the specificity of the assay. We also confirmed the specificity of the binding by coating the anti-penta-HIS surface with His-tagged EYFP (Hytönen, Saeger & Vogel unpublished). Neither core-bradavidin nor chicken avidin showed binding to this surface (data not shown).

## Discussion

We recently characterized a novel biotin-binding protein from the symbiotic bacterium *B. japonicum*, and named the protein bradavidin. It resembles chicken avidin and streptavidin in terms of the biotin-binding properties, but has different immunological properties [9]. This property could be beneficial for development of clinical applications, where bradavidin could be used instead of avidin or streptavidin. In the original study, the evaluation of the biotin-binding properties of bradavidin was carried out by measuring the dissociation rate constants (*k_diss_*) of biotin analogues [9], and the dissociation rate determined at 30°C by radioactive [^3^H]-biotin was very similar for core-bradavidin (*k_diss_* 1.9×10^−4^ s^−1^) and wt bradavidin (*k_diss_* 2.9×10^–4^ s^–1^), but faster than for avidin (*k_diss_* 1.3×10^–7^ s^–1^, extrapolated from the data published in [35]) and streptavidin (*k_diss_* 9.0×10^–6^ s^–1^ extrapolated from the data published in [36]). In contrast, at 50°C, both core-bradavidin and wt bradavidin were just as extreme fluorescent biotin conjugate binders as streptavidin, whereas avidin showed a clearly faster dissociation rate [9]. These experiments led to the conclusion that bradavidin and core-bradavidin do not significantly differ in their ligand-binding properties. However, recent experiments with 1,4,7,10-tetraazacyclododecane-1,4,7,10-tetraacetic acid (DOTA)-conjugated biotin revealed a much lower binding affinity for wt bradavidin in comparison to core-bradavidin (Hytönen and Petronzelli, unpublished results).

In the current study, we solved the X-ray structure of wt bradavidin in order to better understand the function of the full-length protein at structural level. We also conducted ITC experiments both with wt bradavidin and core-bradavidin, which indeed did reveal the lower affinity of wt bradavidin towards biotin in comparison to core-bradavidin. These findings complete our understanding of the ligand-binding properties of wt bradavidin, showing that the C-terminal extension lowers the association rate of the ligand, whereas the extension has no important effect for the dissociation phase, at least in the case of biotin and fluorescently-labelled biotin (Bf560-biotin) [9].

Wt bradavidin has a β-barrel fold typical for avidins. The overall structure of wt bradavidin revealed clear similarities to the known structures of avidins but also unique structural features like the C-terminal extension (see below) and the four Tyr90 residues packing tightly to each other in the center of the tetramer. Tyrosine residues are also found in the subunit interfaces of some known avidin structures, for example in the subunit I-III interface of the AVR4/5 structure [37], but not in the center of the tetramer as in bradavidin (Figure 3). Many of the loops and β-strands of bradavidin also have distinctive structural properties, such as conformation or length. This is especially true for the β-strands and loops near or in contact with the termini of the protein (Figure 1).

Wt bradavidin was crystallized in the presence of HABA, but the azo-dye ligand was not present in the final structure. Instead, the C-terminal amino acids Ser130-Lys137 of each subunit of wt bradavidin folded into the ligand-binding site of the neighbouring subunit (Figure 1). The C-terminal amino acids (SEKLSNTK) were named “Brad-tag” and the affinity of this peptide to core-bradavidin and to other avidins was measured using ITC. We found that Brad-tag binds to core-bradavidin with an affinity (∼25 µM) comparable to that measured between the original Strep-tag and streptavidin [30] (Table 2). Similarly to the binding of other known peptide tags to different avidins (Table 2), the binding of Brad-tag, according to ITC and biosensor analysis, was specific for bradavidin. In ITC analysis, Brad-tag did not interact with other biotin-binding proteins that were analysed, including chicken avidin, streptavidin and rhizavidin. Biosensor analysis initially showed that chicken avidin may have some affinity for surface-immobilized Brad-tagged EGFP. However, our control experiments suggest that this interaction is likely to be non-specific, typical for highly basic avidin. In order to demonstrate that Brad-tag could find use in biotechnological applications, we showed that Brad-tag could be used to concentrate Brad-tag–EGFP protein from cleared cellular lysate. However, the affinity is not yet high enough for efficient purification of fusion proteins and needs to be improved by using mutagenesis or other methods. In our opinion, Brad-tag has potential to be an additional tool within the avidin-biotin technology platform, resembling e.g. Strep-tag [38], a peptide that binds to the ligand-binding site of streptavidin, and Strep-tag II that binds with higher affinity (K_d_ ≈ 1 µM) to an engineered version of streptavidin (streptactin [18]; Table 2).

Our preliminary amino acid sequence comparison of wt bradavidin and its homolog from *Rhodopseudomonas palustris* (Q21816; named rhodavidin [39]) revealed that Trp5, important for the unique conformation of the N-terminus of wt bradavidin, is found at an equivalent position in the sequence of rhodavidin. The high overall similarity for these two proteins (76% sequence identity; Figure 2) indicates potentially similar tertiary structures. More interestingly, the C-terminal Brad-tag sequence (GSEKLSNTK) was found at the C-terminus of rhodavidin but not, to our knowledge, in any other known sequence of the avidin protein family. This suggests a similar mechanism for bradavidin and rhodavidin for the use of the Brad-tag sequence and the question arises as to the biological significance of this sequence. Unfortunately, this is not known yet, although it is clear from our results that the C-terminal extension especially affects the binding of large biotinylated ligands. It has earlier been suggested that the C-terminal residues of the T7-tagged wt streptavidin would compete with weakly bound ligands for the binding site, not least for the reason that the local concentrations of the intrinsic ligands are high [16], [19], [20]. Analogously to wt bradavidin and wt streptavidin, the C-terminal residues of rhodavidin most probably also contribute to the ligand-binding preferences. Because of the much lower affinity for large biotinylated ligands, such as the endogenous biotin carboxyl carrier protein, one could speculate that the biological role of both bradavidin and rhodavidin could be to selectively bind free biotin and, hence, protect the cellular machinery of the host cell. One could also envision the utilization of the ligand-binding preferences of wt bradavidin in biochemical assays.

Even though the biological role of the extended C-termini of wt bradavidin and wt streptavidin may be the same, the structural implementation differs: in wt bradavidin each C-terminus reaches and interacts with the biotin-binding site of the neighbouring subunit (intrinsic, intersubunit ligand), whereas in T7-tagged wt streptavidin the C-terminus interact with the ligand binding site of the same subunit (intrinsic, intrasubunit ligand). The binding mode of the C-termini of wt bradavidin and T7-tagged wt streptavidin is different, too. In wt bradavidin, the key residues for binding to the biotin-binding site are Glu131, Lys132 and Leu133, whereas those of wt streptavidin are Gly151, Asn152 and Pro153 [19]. Moreover, the T7-tag replaces the 13 N-terminal residues of wt streptavidin but no electron density was observed for the tag in the crystal structure [PDB: 2BC3]. Hence, the role of the N-terminus for the structure of wt streptavidin is not fully clear. However, the structure of wt bradavidin reported here shows that the very first N-terminal residues are a unique part of the extended β1-strand; interactions with the first residues after the last β-strand, the β8-strand, affect the folding of the C-terminus, too (Figures 1B, 2).

The ITC-determined dissociation constants (*K_d_*) for Brad-tag to core-bradavidin were 25 µM at 15°C and 26 µM at 40°C. We also studied the characteristics of the interaction between Brad-tag and core-bradavidin by biosensor sandwich assay. Even though this technique was not optimal for accurate determination of the binding constants due to the leakage of the His-tagged protein from the surface (Figure 8A), the 1∶1 binding model fitted to the buffer-subtracted data (Figure 8B) yielded a value of *K_d_* in the micromolar range, which is in good agreement with our ITC-analysis data (Table 3). All in all, the determined dissociation constants were of the same order of magnitude as that for the original Strep-tag to streptavidin (*K_d_* = 37 µM). Although limited, the strength of the Strep-tag-streptavidin interaction was found to be sufficient for affinity purification [30]. The Strep-tag was functional only when attached to the C-terminus of a fusion partner, whereas Brad-tag was functional only at the N-terminus, based on findings made for only one protein, namely EGFP. The terminus-independent affinity tag, Strep-tag II, has also been reported and was discovered by screening a designed peptide array of 400 sequences, but the tag showed lower affinity to streptavidin (*K_d_* ≈ 72 µM) than the original Strep-tag [30]. In order to improve the affinity of Strep-tag II, Voss & Skerra (1997) subjected streptavidin to random mutagenesis resulting in increased affinity (*K_d_* ≈ 1 µM) [18]. Our novel crystal structure of wt bradavidin provides the basis for improving the Brad-tag–core-bradavidin pair, for example, to increase the affinity and other binding properties by rational mutagenesis of core-bradavidin and/or by redesigning the tag.

Several affinity tags have been developed and are widely used in recombinant fusion proteins, e.g. to aid protein purification from crude cell lysates [40], [41] or as tools for probing molecular functions [42]. Affinity tags include a variety of molecules from polypeptides to proteins [40] and several of them bind to avidin, neutravidin or streptavidin (summarized in Table 2). Brad-tag and core-bradavidin utilizing technology would be a valuable addition to this toolbox. Since each tag-based strategy has both advantages and disadvantages, there is a constant need to develop alternative tag-based strategies. These combined with the existing ones may be needed to develop novel methodologies suitable for studies of protein complexes and assemblies. For example, one may envision tagging of several different proteins being an efficient approach to characterize complicated molecular arrangements, such as focal adhesions [43]. On the other hand, by creating circular permutants of bradavidin [44], we could produce bradavidin mutants that would bind more than one type of ligand simultaneously. Moreover, layer-by-layer construction of novel architectures through biological interactions, including avidin-biotin, is an evolving area in the field of optical and electrochemical biosensing [45], where the Brad-tag–core-bradavidin pair could also be used.

In summary, wt bradavidin is a unique member of the avidin protein family having a C-terminal extension, which enters and occupies the ligand-binding site of the neighbouring subunit. The isolated binding epitope has potential to be used as an affinity tag in the field of bio(nano)technology including detection and purification methods as well as construction of novel biomaterials for biosensing and other purposes.

## Materials and Methods

### Protein Expression and Purification

The construction of core-bradavidin and wt bradavidin containing pBVboostFG vectors is described earlier [9]. For Brad-tag–EGFP fusion proteins, the pHis–EGFP plasmid [46, Saeger, Hytönen & Vogel, unpublished] was used as a template in PCR, and primers contained the Brad-tag sequence (Table S1). The amplified PCR-products were extracted from an agarose gel and subcloned into the pET101/D-TOPO® vector according to the manufacturer’s instructions (Invitrogen, Carlsbad, CA, USA). All constructs were confirmed by DNA sequencing (ABI PRISM 3100 Genetic Analyzer, Applied Biosystems).

Core-bradavidin and wt bradavidin were produced in the periplasmic space of *E. coli* BL21-AI cells (Invitrogen) in an active form, as previously described in detail [47]. Similarly, Brad-tag–EGFP fusion proteins were produced in the periplasm of *E. coli* BL21 Star™(DE3) cells (Invitrogen). The pET101/D-based expression vectors were transformed into *E. coli* Star™(DE3) or BL21-AI cells. Fresh transformants were cultured using an orbital shaker with continuous shaking at 28°C in Lysogeny broth (LB) medium, supplemented with 100 µg/ml ampicillin and 0.1% (w/v) glucose for core-bradavidin, 7 µg/ml gentamycin and 0.1% (w/v) glucose for wt bradavidin, and 100 µg/ml ampicillin for Brad-tag–EGFP fusion proteins. When the culture reached an OD_600_ of 0.5, the protein expression was induced by adding 1 mM IPTG (and 0.2% (w/v) L-arabinose for core-bradavidin and wt bradavidin). For Brad-tag–EGFP fusion proteins, bottle cultivation was continued overnight at 28°C, and cells were then collected by centrifugation (4000 g, 10 min, 4°C). For core-bradavidin and wt bradavidin, the pilot-scale fermentation was made with a Labfors Infors 3 (Infors HT, Bottmingen, Switzerland), using the pO_2_ (DO-stat) controlled fed-batch protocol that has been described in detail elsewhere [48].

Core-bradavidin and wt bradavidin were purified in a single step using 2-iminobiotin affinity chromatography (Affiland S. A., Ans-Liege, Belgium) [49]. First, 50 g of cells (wet weight) pelleted from *E. coli* fermentation were suspended in binding buffer (50 mM Na-carbonate, pH 11, containing 1 M NaCl). The suspension was homogenized twice using an EmulsiFlex C3 homogenizator (Avestin Inc., Ottawa, Canada) using homogenizing pressure of 15 000–18 000 psi. After this, cell lysates were centrifuged (15 000 g, 30 min, 4°C). The supernatant was filtered and the pH was measured, and when necessary, adjusted to 10.5 using 10 M NaOH. The crude protein mixture was applied to 2-iminobiotin agarose, which had previously been equilibrated with the binding buffer. This mixture was incubated for one hour on a rolling shaker at 4°C with subsequent centrifugation (3000 g, 10 min, RT) and two washing steps with the binding buffer. Finally, the agarose was transferred to a column and the protein was eluted in one ml fractions with 0.1 M acetic acid (pH 3).

Brad-tag–EGFP fusion proteins containing a His-tag were purified using Ni-NTA metal-affinity chromatography (Qiagen GmbH, Hilden, Germany). First, cells from 500 ml cultivation volume were suspended in lysis buffer (50 mM NaH_2_PO_4_, 300 mM NaCl, 10 mM imidazole, pH 8). Lysozyme (one mg per 100 ml) was added and the cells were sonicated for 5 min (30% duty cycle, 5 s on, 3 s off) on ice. The cellular debris was pelleted by centrifugation (15 000 g, 30 min, 4°C) and the supernatant was filtered using a 0.2 µm filter cloth. Ni-NTA agarose was equilibrated with lysis buffer before mixing with the cleared cellular lysate. The resulting mixture was incubated on a rolling shaker for one hour at 4°C. After this, the mixture was transferred to a column and washed three times with 20 ml of lysis buffer and again three times with 20 ml of wash buffer (50 mM NaH_2_PO_4_, 300 mM NaCl, 20 mM imidazole, pH 8). Finally, the protein was eluted in one ml fractions with elution buffer (50 mM NaH_2_PO_4_, 300 mM NaCl, 250 mM imidazole, pH 8).

Brad-tag–EGFP fusion proteins without a His-tag were purified with core-bradavidin agarose (see below). To prepare the clarified lysate, the cell pellets were suspended in 50 mM Tris-HCl, 100 mM NaCl, pH 7.5. Lysozyme was added to a final concentration of 10 mg/l and the mixture was sonicated and filtered similarly as above.

The purity of all proteins was studied by SDS-PAGE. Cell lysates and eluted proteins were denaturated by heating at 95°C for ten minutes in SDS-PAGE sample buffer containing a reducing agent (β-mercaptoethanol). Samples were run on 15% SDS-PAGE and the gel was stained with Coomassie Brilliant Blue. The amount of EGFP fusion proteins were further investigated by immunoblot analysis. Proteins blotted onto nitrocellulose were detected by incubating with a primary antibody against GFP (Anti-GFP Epitope Tag Polyclonal Antibody, Thermo Scientific) at a 1∶2000 dilution. This was followed by incubation with a secondary antibody - alkaline phosphatase (AP) conjugate (anti-rabbit IgG-AP, Sigma) at a 1∶30000 dilution. Finally, AP was reacted with 5-bromo-4-chloro-3-indolyl phosphate (BCIP) and nitro-blue tetrazolium (NBT) to yield a coloured precipitate.

The protein concentration was determined with a UV/Vis spectrophotometer (NanoDrop 1000 Spectrophotometer, Thermo Scientific, Wilmington, DE, USA) by measuring the absorbance at 280 nm and using an extinction coefficient of 39085 M^–1^cm^–1^ for core-bradavidin and 20525 M^–1^cm^-1^ for EGFP fusion proteins containing Brad-tag and the His-tag.

### Purification of Brad-tag–EGFP Fusion Proteins with Immobilized Core-bradavidin Resin

Core-bradavidin was coupled to NHS-activated resin through amine coupling. First, 4.5 ml of core-bradavidin (0.93 mg/ml) in coupling buffer (10 mM NaHCO_3_, 0.9% NaCl (w/v), pH 7.5) was coupled to Sepharose™ 4 fast flow, NHS-4FF (Affiland, Liège, Belgium). Five ml of resin suspended in isopropanol was centrifuged (3000 g, 10 min, 4°C) and washed twice with 15 ml of cold distilled water and centrifuged again (3000 g, 10 min, 4°C). The supernatant was removed and the protein was added to the resin. The suspension was incubated on a rolling shaker overnight at 4°C followed by incubation on a rolling shaker for one hour at room temperature (RT, 21±1°C). The resin was then centrifuged 3200 g, 10 min, at RT and the supernatant was removed. The protein concentration of the supernatant was measured in order to estimate the amount of resin-bound protein. Next, the resin was washed three times with 14 ml of 10 mM NaHCO_3_, 0.9% NaCl (w/v), pH 7.5, followed by washing three times with 14 ml of PBS buffer, pH 4.0, and again three times with 14 ml of PBS buffer, pH 8.0. Finally, the resin was washed once with 14 ml of PBS buffer, pH 7.4, and stored at 4°C.

Core-bradavidin resin (0.2 ml) was washed with binding buffer (50 mM Tris-HCl, 100 mM NaCl, pH 7.5). Purified Brad-tag–EGFP–His-tag or cleared cellular lysate containing Brad-tag–EGFP was added to the resin and the mixture was incubated on a rolling shaker for one hour at 4°C. After this, the resin was transferred to a column and washed ten times with one ml of binding buffer and again ten times with one ml of wash buffer (50 mM Tris-HCl, 1 M NaCl, pH 7.5). Finally, the protein was eluted in one ml fractions with the elution buffer (50 mM Na-carbonate, 1 M NaCl, pH 11). All fractions were collected and the sample tubes were photographed under UV-light to visualize the elution profile. Samples of fractions were analyzed by 15% SDS-PAGE and the gel stained with Coomassie Brilliant Blue.

To estimate whether the EGFP fusion proteins were concentrated on the resin, 100 µl of the core-bradavidin resin was mixed with 900 µl of binding buffer in the absence and presence of biotin (77 µM). Then, 100 µl of Brad-tag–EGFP–His-tag fusion protein (125 µM) was added and samples were incubated using an orbital shaker with continuous shaking for 10 min. The resin was pelleted by incubation without shaking for 10 min and the sample tubes were photographed under UV-light.

EGFP fusion protein expression and purification were verified by fluorescence. A QuantaMaster™ spectrofluorometer (Photon Technology International, Inc., Lawrenceville, NJ, USA) was used to excite the sample at 485 nm and to detect the emission spectra from 495 nm to 635 nm. The measurements were performed at RT in 50 mM Tris-HCl, 100 mM NaCl, pH 7.5. The emission spectrum of the buffer was subtracted as the baseline.

### Crystallization and X-ray Structure Determination of Wild Type Bradavidin

Suitable conditions for crystallization of bradavidin were found using the Classics™ (Nextal Biotechnology) screen, the vapour diffusion method and sitting drops (1–2 µl) on 96 well plates (Corning Inc.). The protein solution (∼0.4 mg/ml) contained 50 mM sodium acetate (pH 4). Saturated solution of an azo dye HABA (<10 mg/ml) was added to the protein solution in 1∶10 (v/v) ratio, respectively, before crystallization. Bar-like crystals of a typical size of 0.2×0.05×0.05 mm were used for data collection. The crystals were formed at 22°C using 0.7 µl of well solution containing 25% (w/v) PEG 4000, 0.17 M ammonium acetate and 0.08 M sodium acetate (pH 4.6), and 0.8 µl of the protein solution.

X-ray diffraction data were collected at the MAX-lab beam line I911-2 (Lund, Sweden) equipped with a MarCCD detector. The crystal was cryoprotected by adding 0.7 µl of 4 M sodium formate to the crystallization drop just prior to flash-freezing in a 100 K liquid nitrogen stream (Oxford Cryosystem). The collected data was originally processed with Mosflm (7.0.3) [50] using the iMosflm (0.6.1) GUI and scaled with Scala of the CCP4 program suite [51] using the CCP4i GUI [52], and later reprocessed with XDS [53] (see Table 1 for X-ray structure determination statistics).

The initial phase information for structure factors was obtained using the molecular replacement program Phaser [54] within the CCP4i GUI [52]. Multiple search models and ensembles were tested before a solution could be found, finally using homology models as a search ensemble. Shortly, three tetrameric homology models were produced using Modeller [55] of Discovery Studio 2.1 (Accelrys Software Inc.). A structural alignment (data not shown) of the core sequence of bradavidin (Uniprot Q89IH6) and the sequences from X-ray structures of avidin [PDB: 1AVD], streptavidin [PDB: 1MK5] and xenavidin [PDB: 2UYW], respectively, was used for modelling. The models (one model per template structure) were structurally aligned using Pymol [56] and their N- and C-termini were trimmed based on visual checking of the models. Monomers of the aligned structures were then used as a search ensemble in Phaser (two monomers were searched) giving an initial solution with TFZ = 8.5 and LGG = 80 in space group *P*2_1_2_1_2_1_. The initial, incomplete dimeric model of bradavidin was refined using Refmac5 [57] resulting in an *R*_factor_ = 0.513, *R*_free_ = 0.536 and FOM = 0.297 and then used as a search model for the next round of Phaser, where two dimers were searched giving a solution with TFZ = 13.5 and LGG = 246. Refinement of the solution, *i.e.* a tetrameric model of bradavidin, resulted in an *R*_factor_ = 0.449, *R*_free_ = 0.482 and FOM = 0.477. This model was used then, again, as a search model in Phaser now yielding a solution with TFZ = 20.7 and LGG = 320. The model was manually edited/rebuilt using Coot [58] and refined with Refmac5 (*R*_factor_ = 0.420, *R*_free_ = 0.456 and FOM = 0.546) before rebuilding the whole structure with ARP/wARP (starting from an existing model; v. 6.1.1) [59]–[61], finally giving a model of bradavidin that could be finished through further cycles of refinement with Refmac5 and modification/rebuilding with Coot. Solvent atoms and other non-protein atoms were added to the model either with the automatic procedure of ARP/wARP or Coot, or manually in Coot. Few cycles of the refinement in the middle of the structure building was done also with the software suite Phenix [62]. We could not solve the structure without the step-by-step procedure used for molecular replacement described above.

The final structure of wt bradavidin was validated using the inbuilt tools of Coot [58], and using MolProbity [63] of the Phenix software suite [62], before deposition to the Protein Data Bank [64], [65] with PDB entry code 2Y32. The data collection and structure determination statistics are summarized in Table 1.

### Isothermal Titration Calorimetry

The affinity of core-bradavidin towards Brad-tag or D-biotin was measured by isothermal titration calorimetry (ITC). We also analyzed binding between wt bradavidin and D-biotin. The synthetic Brad-tag, SEKLSNTK, was ordered from GenScript (Piscataway, NJ, USA). The purified core-bradavidin or wt bradavidin was dialyzed against 50 mM sodium phosphate (pH 7.0) buffer containing 100 mM NaCl, and Brad-tag and D-biotin were directly dissolved in the same buffer. Samples were degassed with stirring for 5 min and heated to a temperature of 0.5 degrees lower than the measurement temperature by MicroCal™ ThermoVac. The measurements were performed at 15, 25 or 40°C in an isothermal titration calorimetry VP-ITC MicroCalorimeter (GE Healthcare, MicroCal, Northampton, MA, USA) with 10 µl titration aliquots of ligands (Brad-tag, biotin or Brad-tag–EGFP–His-tag) in 30 repeated additions at intervals of 200 s using constant stirring speed 440 rpm. The data were analyzed with Microcal Origin 7.0 (MicroCal LLC, Northampton, MA, USA) software. The observed reaction heats were corrected by subtracting the heat of dilution caused by the titration of the ligand alone into buffer. K_a_, ΔH and n (stoichiometry per subunit) were obtained through non-linear least-squares fit of the corrected reaction heats for each titration step.

### Differential Scanning Calorimetry

The unfolding temperatures of core-bradavidin and wt bradavidin were analyzed by MicroCal™ VP-Capillary DSC System (GE Healthcare, MicroCal, Northampton, MA, USA). Before analysis, the samples were dialyzed against 50 mM sodium phosphate (pH 7.0) buffer containing 100 mM NaCl. The proteins were then diluted to a final concentration of 15 µM. Samples were degassed with stirring for 5 min at 20°C by MicroCal™ ThermoVac. Thermograms were recorded from 20°C to 135°C using scan rate of 120°C/hour. Two parallel measurements of each sample were analysed and each of them were scanned twice. The measurements were conducted in the absence and in the presence of biotin using ∼3∶1 molar ratio of biotin:protein subunit. Microcal Origin 7.0 software was used for data analysis.

### Biolayer Interferometry

Specificity of core-bradavidin binding to Brad-tag–EGFP–His-tag fusion protein was analyzed by biolayer interferometry using ForteBio Octet RED384 instrument (FortéBio, Menlo Park, CA, USA). The instrument was controlled by using Data Acquisition 7.0 software (FortéBio). First, the baseline for anti-Penta-HIS biosensors (FortéBio) in measurement buffer containing 50 mM sodium phosphate (pH 7.0) and 100 mM NaCl was recorded for 200 s. Then, Brad-tag–EGFP–His-tag (0.03 mg/ml) was attached to sensors with 400 s incubation. Following a 10-second washing step in the measurement buffer, the biosensors were moved to a solution containing core-bradavidin (0.06 mg/ml) for 200 s. As a control, 0.06 mg/ml solutions of wt bradavidin, avidin, streptavidin or rhizavidin were studied. Finally, biosensors were exposed to the measurement buffer and the dissociation of bound proteins was measured for 600 s.

The experiment described above was repeated using higher protein concentrations and a slightly longer incubation times: Brad-tag–EGFP–His-tag (0.03 mg/ml) was bound to sensors for 700 s. After washing, the biosensors were then used to study binding of core-bradavidin (0.5 mg/ml), wt bradavidin (1.2 mg/ml), avidin (1.8 mg/ml), streptavidin (1.7 mg/ml) and rhizavidin (2.0 mg/ml) for 200 s. The dissociation of bound proteins in buffer was followed for 1900 s. As a negative control, Brad-tag–EGFP–His-tag coated biosensors, prepared as described above, were incubated in a solution containing core-bradavidin (0.5 mg/ml) and avidin (1.8 mg/ml) in the presence of biotin (3.2 mM for core-bradavidin and 13 mM for avidin). Another control experiment was carried out by using His-tagged EYFP with terminal cysteine residues (Hytönen, Saeger & Vogel unpublished) for the functionalization of the anti-penta-HIS sensors, followed by incubation with various avidins. Yet another control experiment was carried out where plain anti-penta-HIS biosensors were exposed to core-bradavidin (0.5 mg/ml) and chicken avidin (1.8 mg/ml) both in the absence and presence of biotin (3.2 mM for core-bradavidin and 13 mM for avidin). The measurement parameters were as follows: measurement temperature 30°C, stirring speed 1000 rpm and the distance of the tip from the surface 4 mm. Black 96-well plates (Greiner Bio-One GmbH, Frickenhausen, Germany) were used for the biosensor analyses, 1∶1 Langmuir binding model was fitted to the buffer-subtracted binding curve by using Data Analysis 7.0 software (FortéBio). For preparation of the graphs, the raw data was exported from the instrument and processed with MS Excel.

### Miscellaneous Methods

The structure-based sequence alignment was done using Malign of the Bodil software, a modular, multi-platform software package for biomolecular visualization and modeling [66], [67]. ESPript [68] was used for visualization of the sequence alignment. PyMOL (The PyMOL Molecular Graphics System, Version 1.3, Schrödinger, LLC) was used to create all the figures relating to structural representations. ABPS plugin of PyMOL (MG Lerner and HA Carlson. APBS plugin for PyMOL, 2006, University of Michigan, Ann Arbor) was used for electropotential calculations – alternative rotamers were excluded from the calculations. Inkscape 0.47 [69] was used to edit the figures related to structural representations.

## Supporting Information

Figure S1 **Surface properties of wt bradavidin (reported here [PDB: 2Y32]) and T7-tagged wt streptavidin [PDB: 2BC3].** Electropotential maps were calculated using the APBS plugin (MG Lerner and HA Carlson, APBS plugin for PyMOL, 2006, University of Michigan, Ann Arbor) of PyMOL (The PyMOL Molecular Graphics System, Version 1.3, Schrödinger, LLC). Default settings were used and alternative conformers were excluded from the calculations. The views rotated 90 degrees around the x-axis (x90) and y-axis (y90) are also shown.
(TIF)

Figure S2 **Subunit interfaces of wt bradavidin and avidin.** The subunit I-II interface (**A**) and subunit I-III interface (**B**) for wt bradavidin [PDB: 2Y32] (left) and avidin [PDB: 1VYO] (right) are shown. The residues participating to the subunit-subunit interaction are shown as sticks and the carbon atoms coloured as follow: subunit I, blue; II, cyan; and III, magenta.
(TIF)

Figure S3 **Superimposition of the ligand-binding pocket occupying residues of wt bradavidin and wt streptavidin [PDB: 2BC3].** Stereo view. A biotin molecule of chicken avidin structure [PDB: 1AVD] is also shown for the comparison of equivalent moieties. Stick models are shown with colouring of the carbon atoms as follows: wt bradavidin, blue; wt streptavidin, magenta; biotin, white.
(TIF)

Figure S4 **Hydrogen bonding of the side chain nitrogen atom of K132.** A stick model (stereo view) is shown. The carbon atoms of residues from subunit I are shown in blue and from subunit III in magenta. Electron density map (a weighted 2FO-FC map; sigma level 1) around the residues is shown in blue and the putative hydrogen bonds with yellow dashes.
(TIF)

Figure S5 **Structural comparison of peptide tags binding to the ligand-binding site of wt bradavidin and streptavidin.** Carbon atoms of wt bradavidin and streptavidin are shown in blue and green, respectively. The PDB entry codes are shown in brackets.
(TIF)

Figure S6 **Brad-tag titrations to control proteins by ITC.** Thermograms of measurements performed at 40°C for (**A**) avidin, (**B**) streptavidin and (**C**) rhizavidin are shown. No detectable binding of Brad-tag to these proteins is seen.
(TIF)

Figure S7 **Interaction between various avidin proteins and Brad-tag analyzed by biolayer interferometry.** (**A**) Anti-Penta-HIS biosensors were coated with Brad-tag–EGFP–His-tag fusion protein (step 1, arrow in the graph). After a brief wash (10 s) in measurement buffer, biosensors were incubated with a series of different proteins: core-bradavidin (0.5 mg/ml), wt bradavidin (1.2 mg/ml), avidin (1.8 mg/ml), streptavidin (1.7 mg/ml) and rhizavidin (2.0 mg/ml) and a buffer as a control (step 2). Binding of core-bradavidin was detected and a slight increase in the signal for avidin as well. Finally, biosensors were exposed to buffer and the bound proteins started to dissociate (step 3). (**B**) The measured raw data for buffer is subtracted from the raw data measured for different proteins. (**C**) As a control measurement, core-bradavidin (0.5 mg/ml) and chicken avidin (1.8 mg/ml) were measured in the presence of biotin (3.2 mM for core-bradavidin and 13 mM for avidin). The data where the effect of the used measurement buffer is subtracted is shown. (**D**) As another control measurement, core-bradavidin (0.5 mg/ml) and chicken avidin (1.8 mg/ml) in the absence and presence of biotin (3.2 mM for core-bradavidin and 13 mM for avidin) were incubated with plain anti-penta-HIS biosensors.
(TIF)

Table S1 **Sequences of primers used in PCR reactions.**
(DOC)
