# Supplementary figures and images for: Structure of Bradavidin – C-Terminal Residues Act as Intrinsic Ligands
Source: PLoS One. 2012 May 4;7(5):e35962. doi: 10.1371/journal.pone.0035962 (PMC3344845; doi:10.1371/journal.pone.0035962)

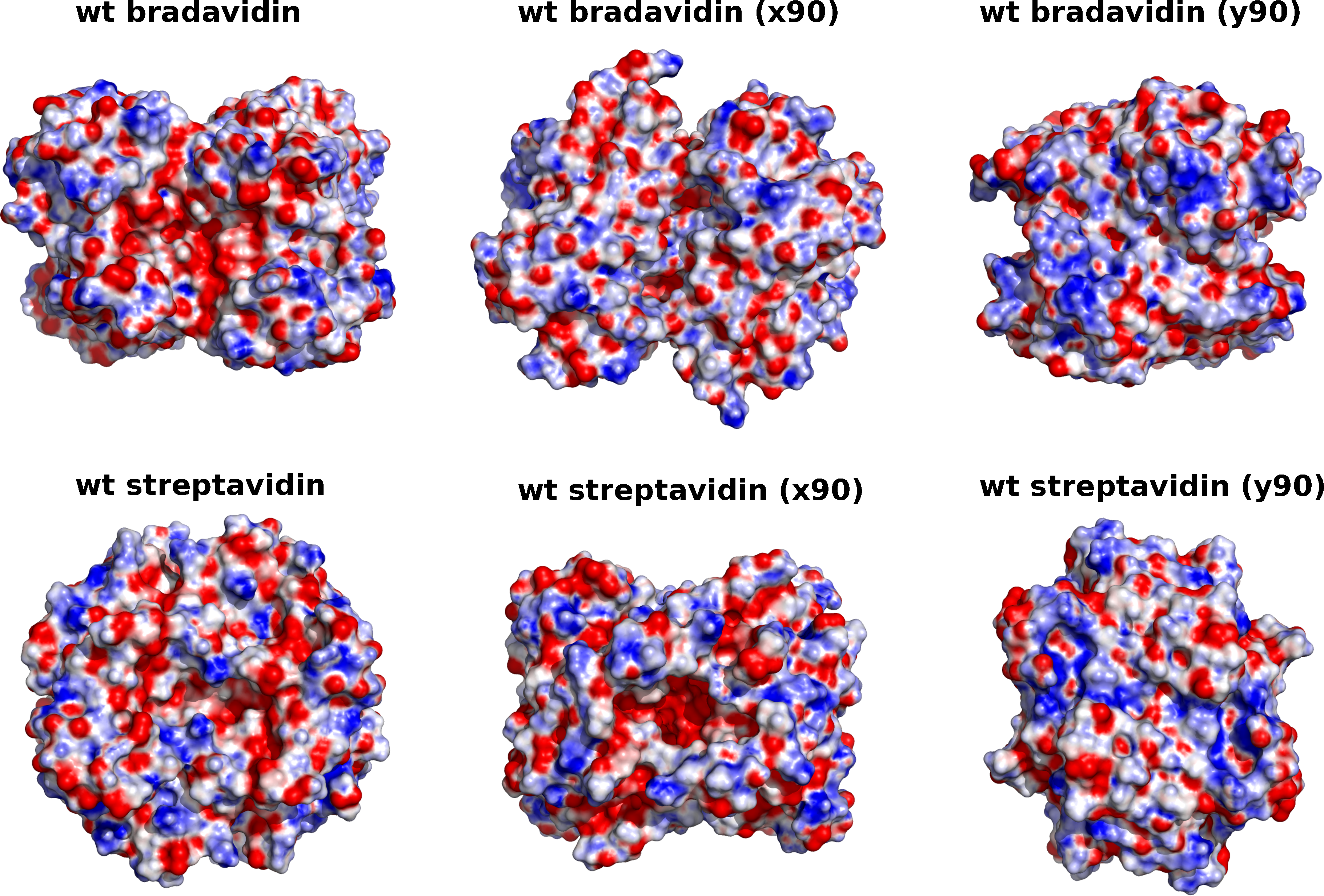

Supplement: Figure S1 — Surface properties of wt bradavidin (reported here [PDB: 2Y32]) and T7-tagged wt streptavidin [PDB: 2BC3]. Electropotential maps were calculated using the APBS plugin (MG Lerner and HA Carlson, APBS plugin for PyMOL, 2006, University of Michigan, Ann Arbor) of PyMOL (The PyMOL Molecular Graphics System, Version 1.3, Schrödinger, LLC). Default settings were used and alternative conformers were excluded from the calculations. The views rotated 90 degrees around the x-axis (x90) and y-axis (y90) are also shown. (TIF) [file pone.0035962.s001.tif]

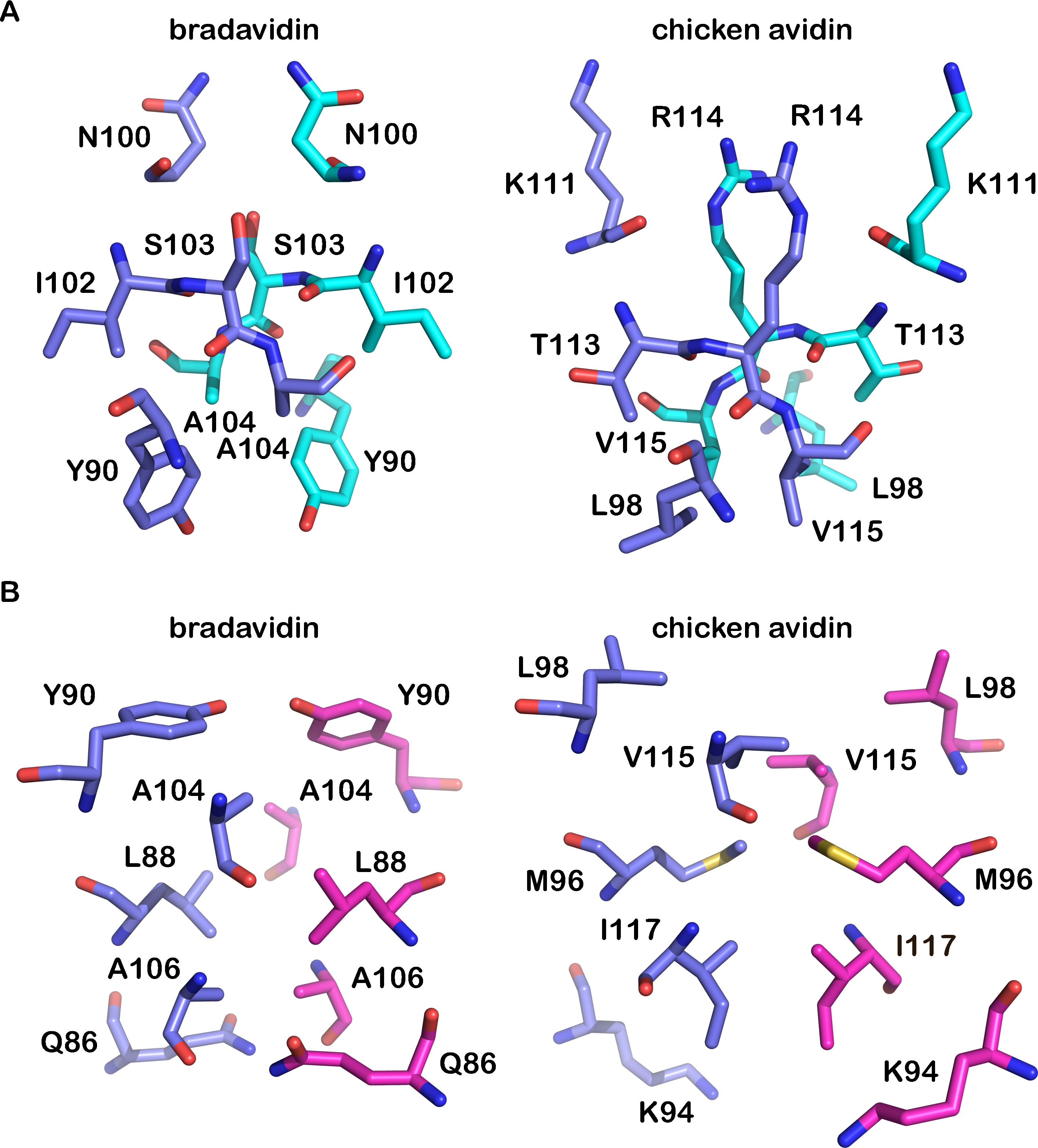

Supplement: Figure S2 — Subunit interfaces of wt bradavidin and avidin. The subunit I-II interface (A) and subunit I-III interface (B) for wt bradavidin [PDB: 2Y32] (left) and avidin [PDB: 1VYO] (right) are shown. The residues participating to the subunit-subunit interaction are shown as sticks and the carbon atoms coloured as follow: subunit I, blue; II, cyan; and III, magenta. (TIF) [file pone.0035962.s002.tif]

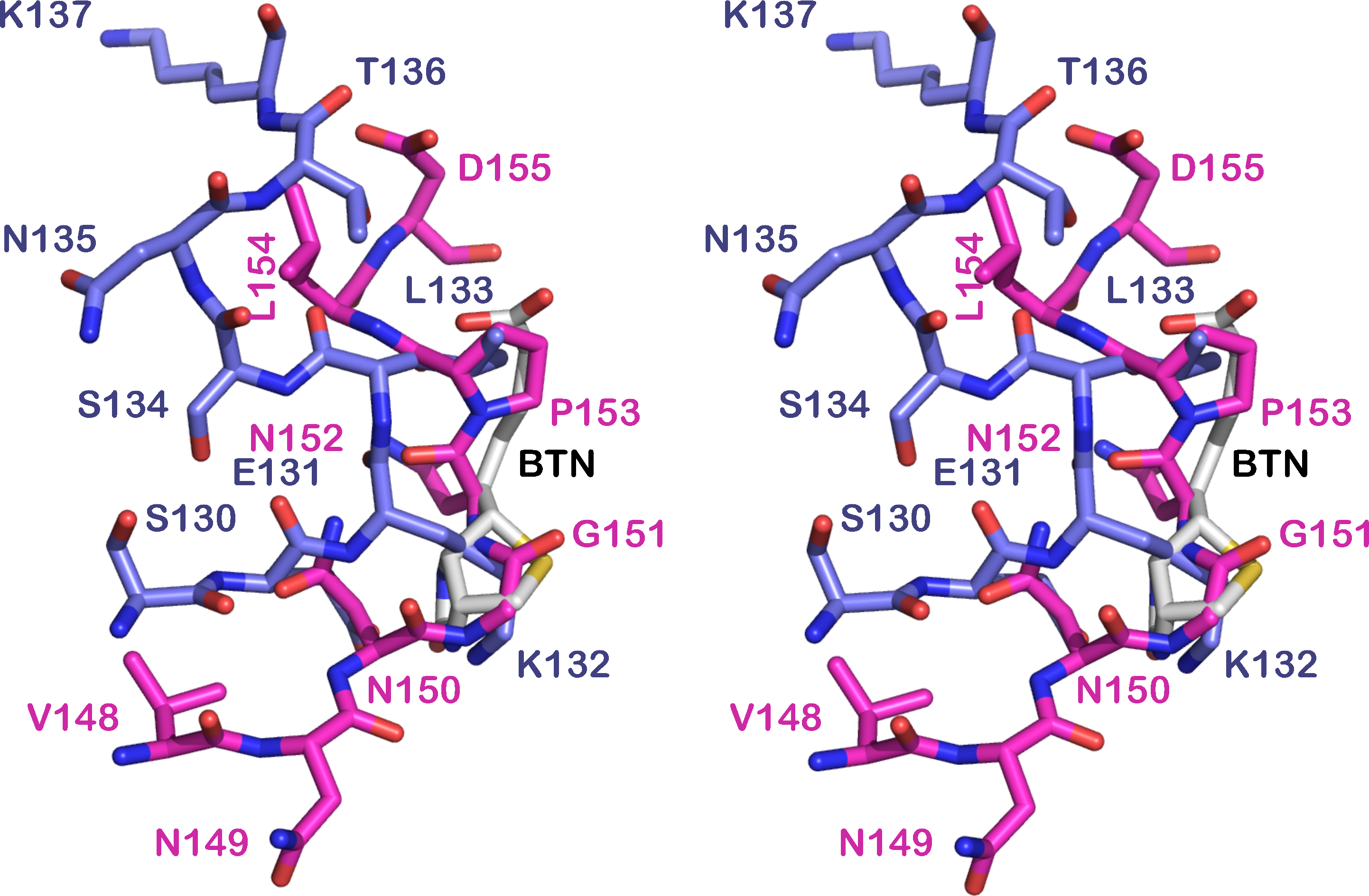

Supplement: Figure S3 — Superimposition of the ligand-binding pocket occupying residues of wt bradavidin and wt streptavidin [PDB: 2BC3]. Stereo view. A biotin molecule of chicken avidin structure [PDB: 1AVD] is also shown for the comparison of equivalent moieties. Stick models are shown with colouring of the carbon atoms as follows: wt bradavidin, blue; wt streptavidin, magenta; biotin, white. (TIF) [file pone.0035962.s003.tif]

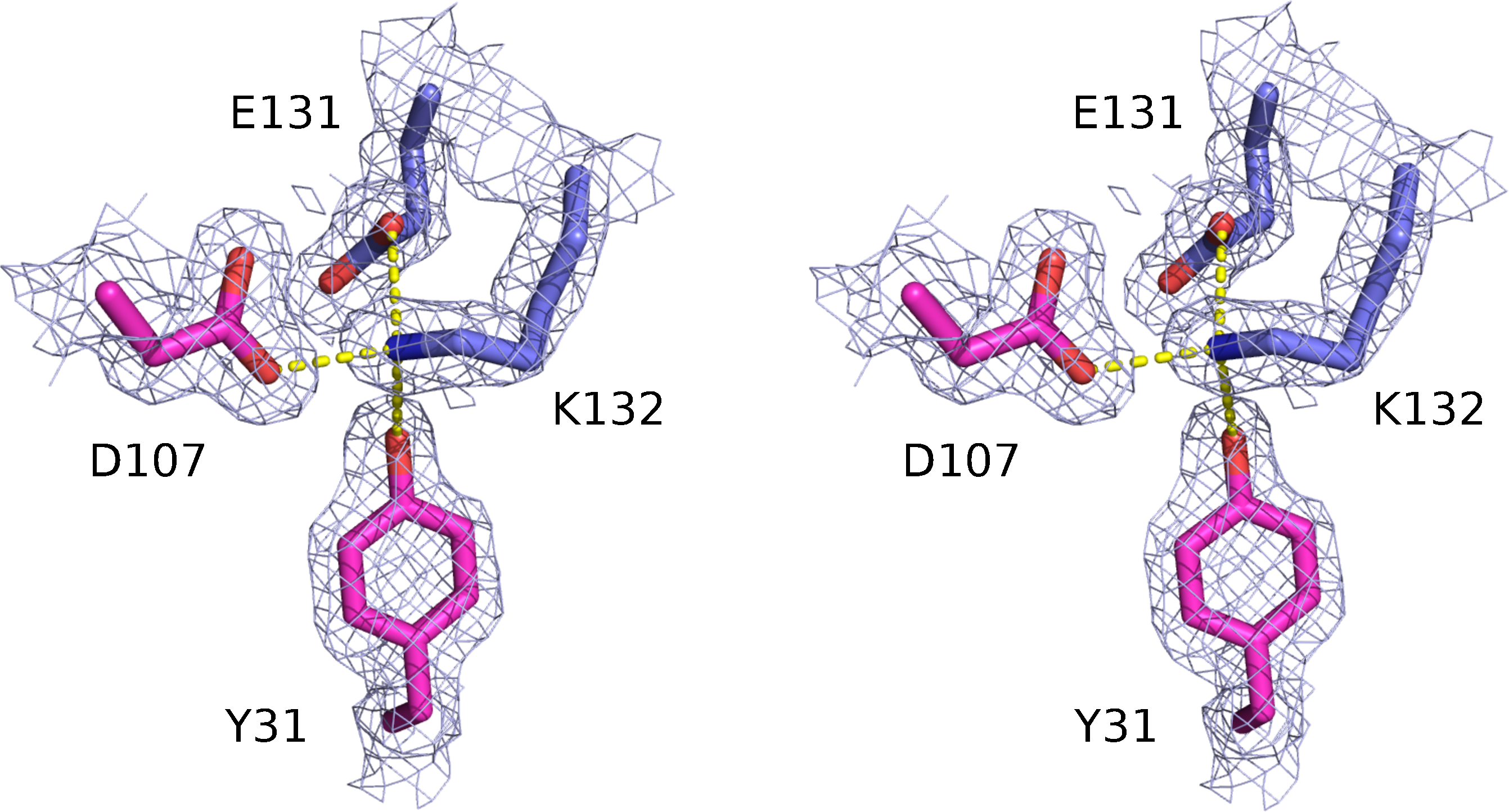

Supplement: Figure S4 — Hydrogen bonding of the side chain nitrogen atom of K132. A stick model (stereo view) is shown. The carbon atoms of residues from subunit I are shown in blue and from subunit III in magenta. Electron density map (a weighted 2FO-FC map; sigma level 1) around the residues is shown in blue and the putative hydrogen bonds with yellow dashes. (TIF) [file pone.0035962.s004.tif]

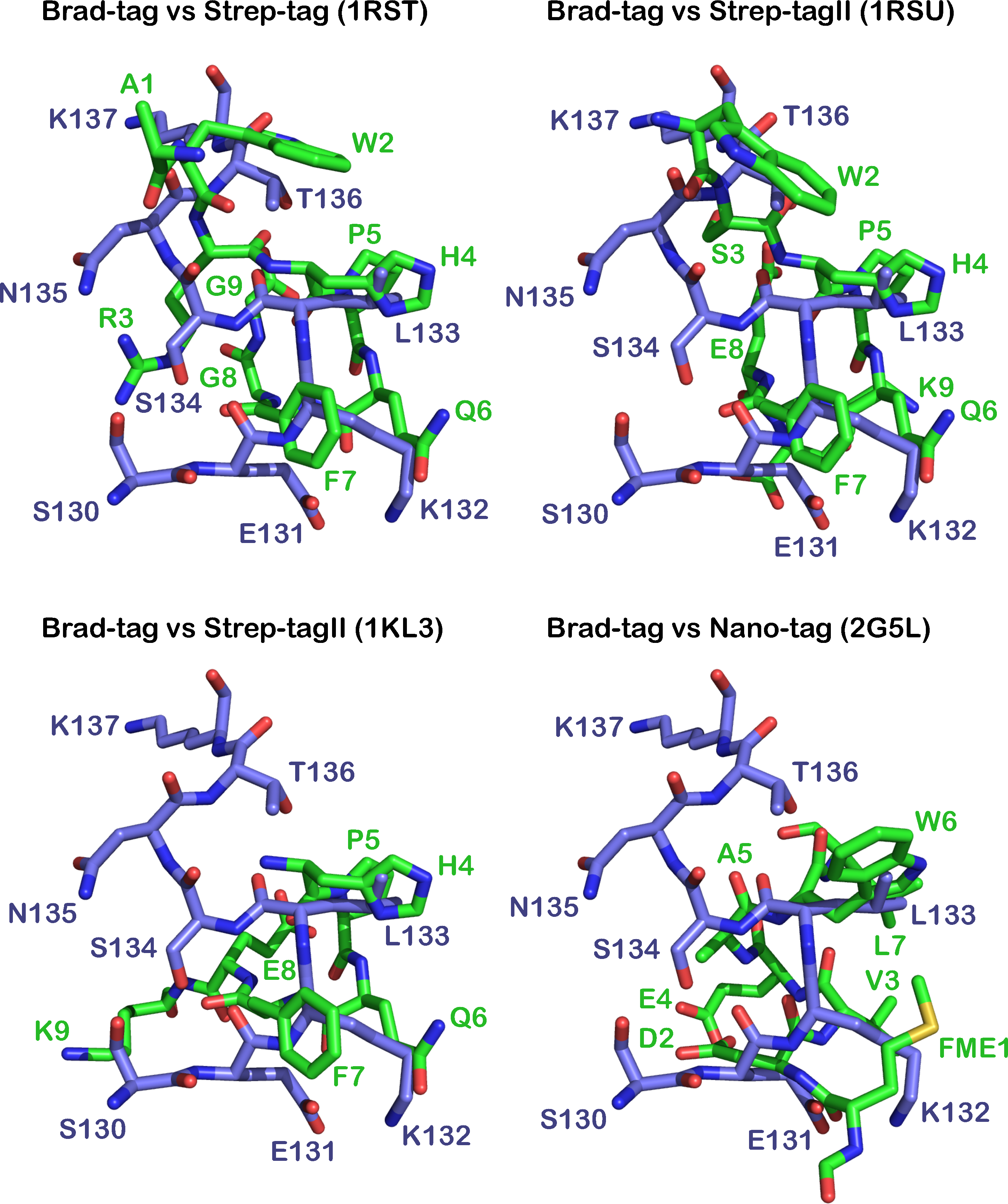

Supplement: Figure S5 — Structural comparison of peptide tags binding to the ligand-binding site of wt bradavidin and streptavidin. Carbon atoms of wt bradavidin and streptavidin are shown in blue and green, respectively. The PDB entry codes are shown in brackets. (TIF) [file pone.0035962.s005.tif]

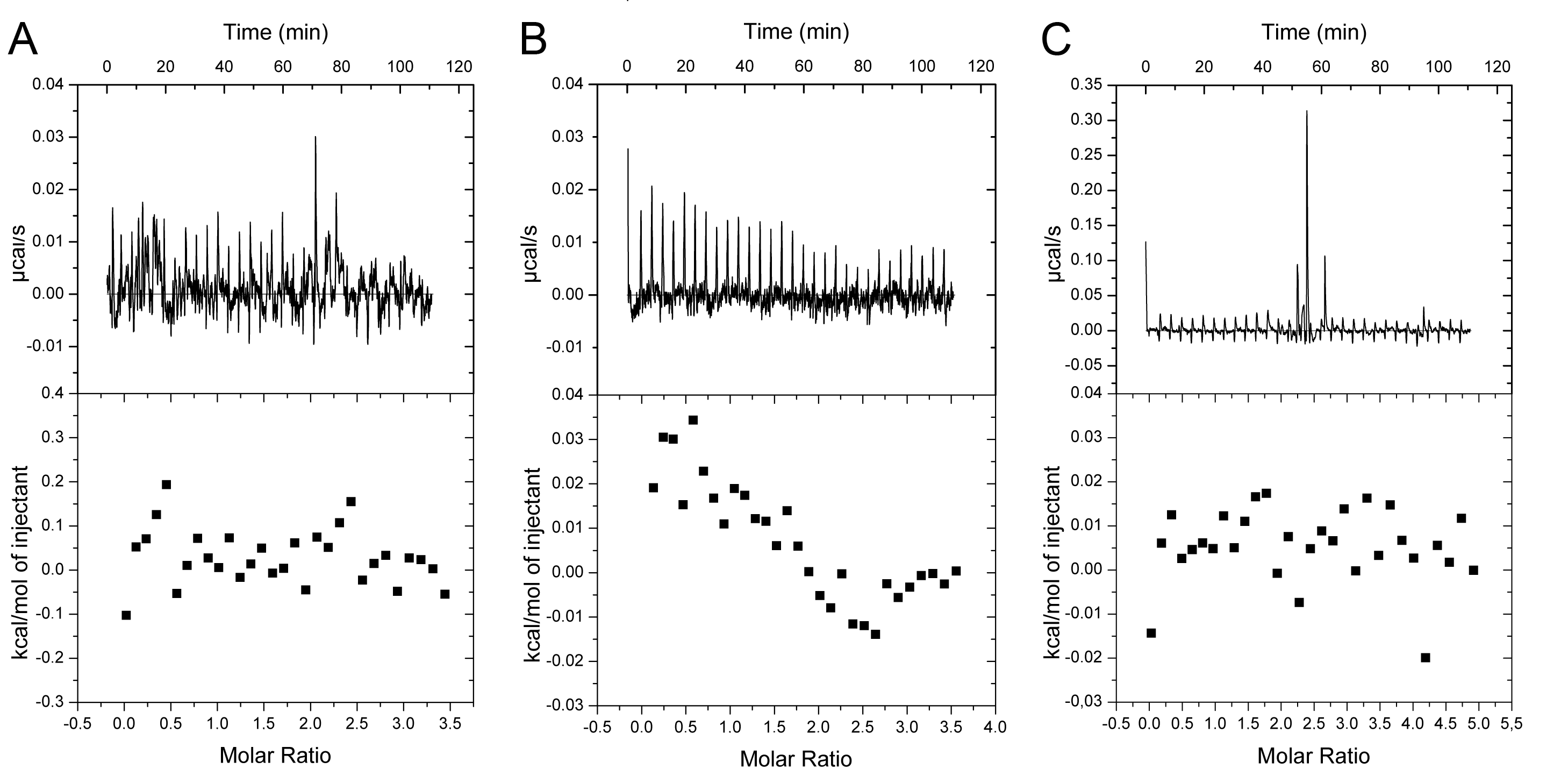

Supplement: Figure S6 — Brad-tag titrations to control proteins by ITC. Thermograms of measurements performed at 40°C for (A) avidin, (B) streptavidin and (C) rhizavidin are shown. No detectable binding of Brad-tag to these proteins is seen. (TIF) [file pone.0035962.s006.tif]

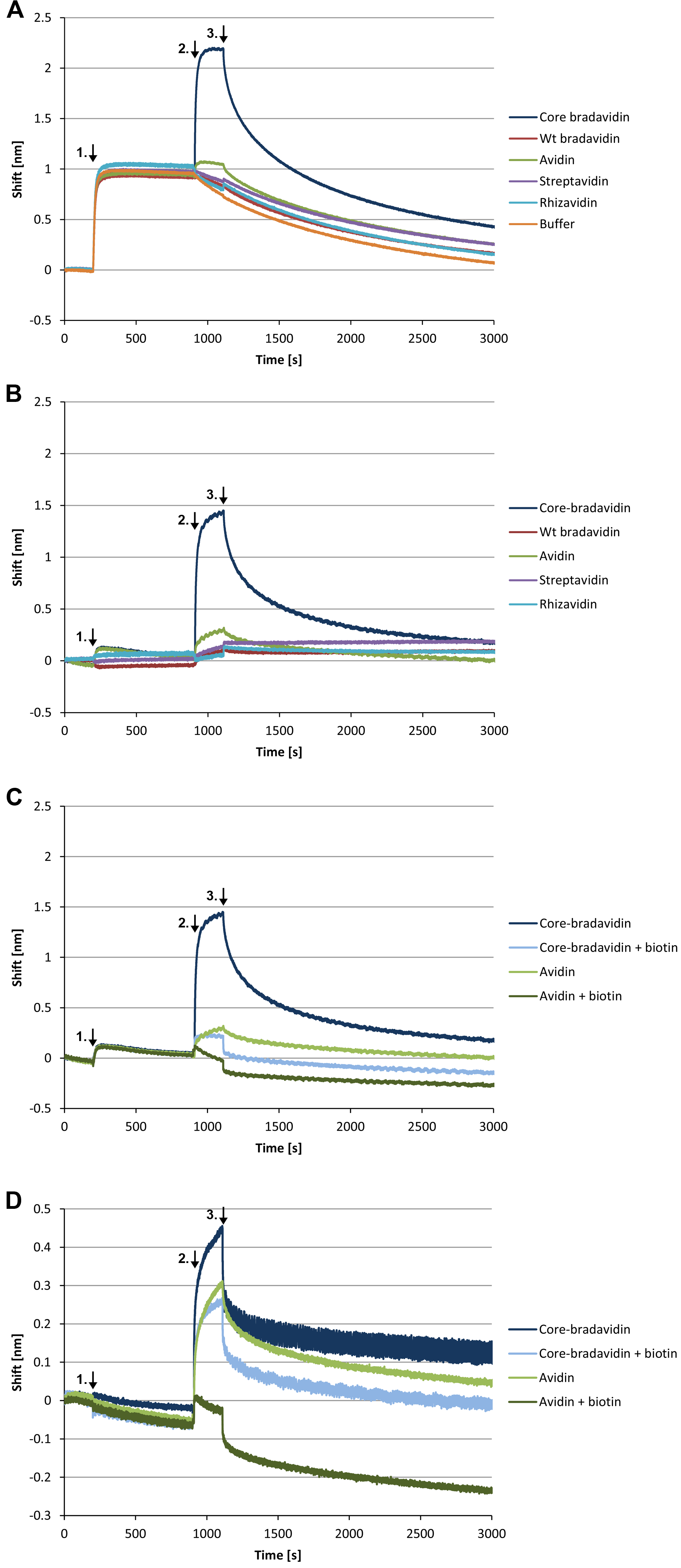

Supplement: Figure S7 — Interaction between various avidin proteins and Brad-tag analyzed by biolayer interferometry. (A) Anti-Penta-HIS biosensors were coated with Brad-tag–EGFP–His-tag fusion protein (step 1, arrow in the graph). After a brief wash (10 s) in measurement buffer, biosensors were incubated with a series of different proteins: core-bradavidin (0.5 mg/ml), wt bradavidin (1.2 mg/ml), avidin (1.8 mg/ml), streptavidin (1.7 mg/ml) and rhizavidin (2.0 mg/ml) and a buffer as a control (step 2). Binding of core-bradavidin was detected and a slight increase in the signal for avidin as well. Finally, biosensors were exposed to buffer and the bound proteins started to dissociate (step 3). (B) The measured raw data for buffer is subtracted from the raw data measured for different proteins. (C) As a control measurement, core-bradavidin (0.5 mg/ml) and chicken avidin (1.8 mg/ml) were measured in the presence of biotin (3.2 mM for core-bradavidin and 13 mM for avidin). The data where the effect of the used measurement buffer is subtracted is shown. (D) As another control measurement, core-bradavidin (0.5 mg/ml) and chicken avidin (1.8 mg/ml) in the absence and presence of biotin (3.2 mM for core-bradavidin and 13 mM for avidin) were incubated with plain anti-penta-HIS biosensors. (TIF) [file pone.0035962.s007.tif]
