# Supplementary material for: Structure of Bradavidin – C-Terminal Residues Act as Intrinsic Ligands
Source: PLoS One. 2012 May 4;7(5):e35962. doi: 10.1371/journal.pone.0035962 (PMC3344845; doi:10.1371/journal.pone.0035962)
Supplement: Table S1 — Sequences of primers used in PCR reactions. (DOC) [file pone.0035962.s008.doc]

| Primer | Sequence |
| --- | --- |
| 5’ Brad-tag_GFP | 5'-CACCATGTCA GAAAAACTGT CAAATACAAA AAGCAAGGGC GAGGAG-3' |
| 5’ GFP | 5'-CACCATGAGC AAGGGCGAGG AG-3' |
| 3’ GFP_Stop | 5'-TCAAGTGATC CCGGCGGC-3' |
| 3’ GFP | 5'-AGTGATCCCG GCGGC-3' |
| 3’ GFP_Brad-tag_Stop | 5'-TCATTTTGTA TTTGACAGTT TTTCTGAAGT GATCCCGGCG GC-3' |
| 3’ GFP_6xHis_Brad-tag_Stop | 5’-TTATCATTTT GTATTTGACA GTTTTTCTGA ATGGTGATGG TGATGATGAC CGGTAC-3’ |
